# Tension of plus-end tracking protein Clip170 confers directionality and aggressiveness during breast cancer migration

**DOI:** 10.1038/s41419-022-05306-6

**Published:** 2022-10-08

**Authors:** Yunfeng Hu, Qiu Xie, Xiang Wu, Weizhen Liu, DongFang Li, Chen Li, WangXing Zhao, LinLin Chen, Zihui Zheng, GuangMing Li, Jun Guo

**Affiliations:** 1 School of Medicine & Holistic Integrative Medicine, Nanjing University of Chinese Medicine, Nanjing, 210023 Jiangsu P. R. China; 2 Key Laboratory of Drug Target and Drug for Degenerative Disease, Nanjing University of Chinese Medicine, Nanjing, 210023 Jiangsu P. R. China; 3 Department of Anesthesiology, The Affiliated Hospital of Medical School, Ningbo University, Ningbo, 315040 P. R. China; 4 Department of Anesthesiology, Huaian First People’s Hospital, Nanjing Medical University, Huaian, Jiangsu 223001 P. R. China

**Keywords:** Breast cancer, Phosphoproteins

## Abstract

The microtubule (MT) plus-end binding protein Clip170 is associated closely with breast cancer invasion and migration. In this study, Clip170 tension observed by a newly designed cpstFRET tension probe was suggested to be positive related to breast cancer aggressiveness, which could be regulated by α-tubulin detyrosination-induced MT disassembly. Clip170 phosphorylation induced by Ribosomal protein S6 kinase (RSK) could also increase its tension and promote the conversion of a discrete comet-like Clip-170 distribution into a spotty pattern during cancer metastasis. Heightened Clip170 tension was correlated with the formation of cortactin-associated filopodia and lamellipodia, and then promoted invasion and metastasis both in vitro and in vivo. Meanwhile, Clip170 tension enhanced at the leading edge in directional migration, accompanying with IQGAP1 subcellular distribution variation. Our work indicates that the malignancy and directionality during breast cancer migration depend on the magnitude and polarization of Clip170 tension, and we suggest Clip170 tension as a new potential drug target for breast cancer therapy.

## Introduction

Breast cancer is the most frequent malignancy with highest prevalence and death rate in women [1]. Nearly 40% of breast cancer will develop into metastatic cancer, with poor prognosis and high recurrence rate, for which appropriate therapeutic strategies are lacking [2, 3]. In recent years, intracellular mechanical activities come to prominence in cancer research [4, 5]. Therefore, it is necessary to identify the mechanical mechanisms underlying breast cancer invasion and metastasis.

Based on the previous study, the aggressiveness of cancer cell is closely correlated with the microtubule (MT) force [6], which relies on the MT structure dynamic variation [7] and movement of motor proteins [8]. It is widely accepted that the MT-associated motor protein dynein generates protrusive activity at the front (anterior), coupled with microfilament (MF)-associated motor protein myosin generating contraction at the sides and rear (posterior), providing the major driving forces for migration [9, 10]. MT force promotes microtubule extension to the cell cortex and cytomembrane, which relies on plus-end tracking proteins (+TIPs) [11]. However, whether +TIPs influence breast cancer aggressiveness via MT-associated mechanical and potential regulatory mechanism remains unclear.

MT force transmission to the cell cortex and cytomembrane relies on scaffold proteins. +TIPs are considered as significant scaffold proteins between MTs and the cell cortex during cancer progression [12]. The CAP-Gly domain-containing linker protein 1 (Clip170) is the first +TIP protein to be demonstrated to control MT dynamics, providing a bridge to the cell cortex [13]. Together with flanking three serine-rich regions, the tandem N-terminal MT-binding glycine-rich cytoskeleton-associated protein (CAP-GLY) domain [14] directly binds to the freshly polymerized distal ends of growing MTs [15, 16], guiding the direction of MT extension, then destabilizes focal adhesions and enhances cell motility [17]. Meanwhile, Clip170 links the MT-binding subunit of dynactin p150^Glued^ via its zinc-finger motif to regulate dynactin/dynein complex formation, thereby accelerating cancer cell polarization and the MT rescue rate [18]. In addition, Clip170-associated MT force transmission could be regulated by other scaffold proteins. Previous study reports that MTs and MFs can be mechanistically coupled by Clip170-IQGAP complex at the leading edge, inducing microtentacles formation and degradation of the collagen-rich extracellular matrix [19].

Clip170 tracking on MT could be regulated with other tubulin C-terminus bound enzymes during cancer progression [20, 21]. Vasohibin (VASH) 1/2 catalyzes α-tubulin to form detyrosinated-tubulin [22, 23] and tubulin tyrosine ligase (TTL) can add a tyrosine to the C-terminus of detyrosinated α-tubulin to form tyrosinated-tubulin [24, 25]. The increased levels of α-tubulin detyrosination accelerate MT depolymerization, enhancing MT dynamics and assembly speed [26, 27]. MT instability induced by α-tubulin detyrosination leads to incorrect kinetochore-MT attachments and chromosomal instability, which are considered as hallmarks of cancers and are implicated in tumor evolution and metastasis [28]. Meanwhile, α-tubulin detyrosination promotes epithelial-mesenchyme transition (EMT) and increases the number of microtentacles that penetrate endothelial layers to facilitate tumor cell reattachment [29]. However, whether the MT structure disassembly caused by α-tubulin detyrosination regulates Clip170’s mechanical properties and breast cancer aggressiveness are unknown.

In the present study, we investigate the role of Clip170 tension during breast cancer invasion and migration, and focus on the mechanism underlying MT force transmission regulated by Clip170 phosphorylation and α-tubulin tyrosination. Using Clip170 tension probe based on fluorescence resonance energy transfer (FRET), the mechanical transmission model mediated by Clip170 tension in cancer invasion and migration could be studied visually, and could provide the theoretical basis for breast cancer treatment via reducing cancer cell mobility.

## Materials And Methods

### Cell culture

Four kinds of breast cancer cell lines, MDA-MB-231, SKBR3, MDA-MB-468, and MCF7, were purchased from Shanghai Cell Bank Type Culture Collection Committee (CBTCCC, Shanghai, China). MCF7, SKBR3, and MDA-MB-468 cells were cultured in Dulbecco’s Modified Eagle’s Medium (Gibco, Grand Island, NY, USA) containing 10% fetal bovine serum (FBS, Gibco) and antibiotics (100 U/mL streptomycin and penicillin). All cells were grown under humidified air containing 5% CO_2_ at 37 °C. MDA-MB-231 cells were cultured in Leibovitz L-15 Medium (Gibco), containing 10% FBS and antibiotics (100 U/mL streptomycin and penicillin).

### Reagents and antibodies

Clip170 polyclonal antibodies (23839-1-AP, western blotting 1:1000), N-cadherin polyclonal antibodies (22018-1-AP, western blotting 1:1000), CFP Polyclonal antibody (17192-1-AP, western blotting 1:1000), Beta Actin Polyclonal antibody (20536-1-AP, western blotting 1:1000), E-cadherin polyclonal antibodies (20874-1-AP, western blotting 1:1000), ribosomal protein S6 kinase A1 (RSK) (RPS6KA1) polyclonal antibodies (16463-1-AP, western blotting 1:1000), Phospho-RPS6KA1 (Ser380) polyclonal antibodies (28983-1-AP, western blotting 1:1000), IQ motif containing GTPase activating protein 1 (IQGAP1) polyclonal antibodies (22167-1-AP, western blotting 1:1000) and IQGAP2 polyclonal antibodies (55189-1-AP, western blotting 1:1000) were obtained from Proteintech Group (Rosemont, IL, USA). Antibodies against vimentin (D21H3, western blotting 1:1000) and cortactin (H222, western blotting 1:1000) were purchased from Cell Signaling Technology (Danvers, MA, USA). Anti-α-tubulin antibodies (BM3885, western blotting 1:2000) were purchased from Boster (Shanghai, China). Anti-detyrosinated alpha-tubulin (ab48389, western blotting 1:500) was obtained from Abcam (Cambridge, UK). Rhodamine [tetramethylrhodamine (TRITC)]-conjugated goat anti-rabbit IgG (H + L) (ZF-0316, IF 1:100), fluorescein-conjugated goat anti-mouse IgG (H + L) (ZF-0312, IF 1:100), fluorescein-conjugated goat anti-rabbit IgG (H + L) (ZF-0311, IF 1:100), and rhodamine (TRITC)-conjugated goat anti-mouse IgG (H + L) (ZF-0313, IF1:100) were obtained from Zsgb-Bio (Beijing, China).

Recombinant human SDF-1β (C-X-C motif chemokine ligand 1 (CXCL12)) and transforming growth factor β (TGFβ) were obtained from Peprotech (Rocky Hill, NJ, USA). The RSK inhibitor BRD7389 was purchased from MedchemExpress (Monmouth Junction, NJ, USA). 4′, 6-Diamidino-2-phenylindole (DAPI) was obtained from Beyotime Biotechnology (Jiangsu, China). Phalloidin was purchased from Solarbio (Shanghai, China).

### Western blotting

All cells were dissolved in radioimmunoprecipitation assay (RIPA) lysis buffer (Beyotime Biotechnology) supplemented with phenylmethylsulfonyl fluoride (PMSF) (Roche, Basel, Switzerland) and a protease inhibitor cocktail (Millipore Sigma, St. Louis, MO, USA). Cells lysates were denatured by boiling in loading buffer. The extracted total proteins were separated via SDS-PAGE and transferred to nitrocellulose membranes, which were incubated in 5% non-fat milk and blocked for 1 h. The membranes were then incubated with specific antibodies overnight at 4 °C. After washing three times, the membranes were incubated with secondary antibodies for 2 h. The enhanced chemiluminescence (ECL) chromogenic substrate was used to visualize the immunoreactive protein bands, and the protein band intensities were quantified using densitometry (Quantity One; Bio-Rad, Hercules, CA, USA). α-tubulin was set as control.

### Immunofluorescence analysis

Cells were removed from their medium and washed three times with phosphate-buffered saline (PBS). Paraformaldehyde solution (4%) was used to fix cells for 30 min at room temperature. After that, the cells were treated with 0.1% Triton X-100 in PBS for 10 min at room temperature, blocked with 4% bovine serum albumin/PBS for 30 min at room temperature, and incubated with primary antibodies overnight at 4 °C. After washing with PBS three times, the cells were incubated with secondary antibodies for 1 h in the dark at 37 °C. DAPI was used to stain the nuclei. The images with FITC and TRITC were imaged with a 63× objective lens under the constant exposure (250 ms) and gain (1.0). The DAPI were imaged with a 63× objective lens under the constant exposure (50 ms) and gain (1.0). The change in the fluorescence value was monitored under a Leica DMI8 inverted fluorescence microscope (Leica, Wetzlar, Germany).

### Plasmid construction and small interfering RNA (siRNA) design

The mEmerald-CLIP170-C-18 (#54043) was purchased from Addgene (Watertown, MA, USA). pCMV3-TTL (HG23280-UT) and Pcmv3-Flag-IQGAP2 plasmids were obtained from Sino Biological (Beijing, China). The FRET-based force probes were constructed using a NovoRec PCR Seamless Cloning Kit (Thermo Fisher Scientific, Waltham, MA, USA) and restriction enzyme cloning. We constructed the Clip170 fluorescence sensor with circularly permutated enhanced cyan fluorescent protein (eCFP) and enhanced yellow fluorescent protein (eYFP) [mTurquoise2-7aa-super (s) YFP2 circularly permuted stretch-sensitive FRET (cpstFRET)], which was inserted between aa 350 and aa 351 of Clip170. The S311A, S311D, S-A, and S-D mutations were constructed using KOD-Plus-Neo (TOYOBO, Osaka, Japan). All the plasmids were prepared using Endo-Free Plasmid Mini Kits (Omega Bio-Tek, Norcross, GA, USA).

The small interfering RNAs (siRNAs) targeting *TTL*, *IQGAP1*, *IQGAP2*, and *RSK*, and the negative control siRNA (NC) were designed by Genepharma (Shanghai, China)

*RSK1* sense: 5′-CCAUGACACUGAUUCUGAATT-3′

Antisense: 5′-UUCAGAAUCAGUGUCAUGGTT-3′

*TTL* sense: 5′-CAGCCACCAAUCAGUAACUTT-3′

Antisense: 5′-AGUUACUGAUUGGUGGCUGTT-3′

*IQGAP1* sense: 5′-GGCAAUUUAAAUGACCCAATT-3′

Antisense: 5′-UUGGGUCAUUUAAAUUGCCTT-3′

*IQGAP2* sense: 5′-GGUUAAUGCUCAAAUUCAATT-3′

Antisense: 5′-UUGAAUUUGAGCAUUAACCTT-3′

NC sense: 5′-UUCUCCGAACGUGUCACGUTT-3′

Antisense: 5′- ACGUGACACGUUCGGAGAATT-3′

### Cpst-FRET probe transfection

To control ectogenic cpstFRET-Clip170 probe and Clip170 mutation probes at the same levels in different groups, cells were transfected under the same condition in WT and mutation groups. 1 μg cpstFRET-Clip170 probe or Clip170 mutation probes and 3 μL transfection reagent (Lip2000 Transfection reagent, Thermo Fisher) were diluted with 100 μL Opti-MEM medium (Thermo Fisher). The diluted transfection reagent was subsequently mixed with the diluted nucleotide and incubated for 15 min at room temperature. The mixture was then added to a 6-well plate at a density of 2 ×10^5^ cells/well, immediately at room temperature, after which the cells were incubated at 37˚C. The subsequent experiments were performed at 48 h after transfection. After that, the CFP expression levels were detected to verify the transfection efficiency of Clip170 at same levels in different groups.

### Stable cell lines and xenograft study

The Clip170-cpstFRET, Clip170-S311A-cpstFRET, and Clip170-S311D-cpstFRET probes were cloned into the PCDH vector. Firstly, HEK 293T cells were cultured in a cell culture plate until they reached 70–80% confluence. Further, cells were transfected with 3 μg cpstFRET-Clip170 probe or Clip170 mutation probes(PCDH vector), along with helper plasmids, PSPAX2 (2.25 μg) and PM2G (0.9 μg). The media of HEK-293T cells were collected 48 h after transfection, and the virus was harvested. The MDA-MB-468 cells were seeded on a 6-well plate at a density of 2 ×10^5^ cells/well and infected with 1 × 10^7^ TU/mL for 48 h. The stable transfected cells were screened using puromycin (2 μg/mL) [30]. The stable MDA-MB-468 cell lines are used in Fig. 5 and Fig. 6.

Animal studies were performed according to the institutional guidelines approved by Institutional Animal Care and Use Committee of Nanjing University of Chinese Medicine. Thirty-six female BALB/c nude mice (six weeks old) were obtained from the Institute of Comparative Medicine of Yangzhou University (Yangzhou, China) and maintained under specific pathogen-free conditions at Nanjing University of Chinese medicine. The mice were randomly assigned into six groups: Clip170-wild-type (WT)-tail vein injection, Clip170-S311A-tail vein injection, Clip170-S311D-tail vein injection, Clip170-WT-orthotopic implantation, Clip170-S311A-orthotopic implantation, and Clip170-S311D-orthotopic implantation.

For the orthotopic implantation groups, 1 × 10^6^ screened cells were resuspended in 50 μL of medium containing 20 μL of Matrigel and injected into the mouse fat pads. For the tail vein injection groups, 1 × 10^6^ screened cells were resuspended in 50 μL of medium and injected into the tail vein. Cancer progression was monitored and quantified using a Quantum GX and Spectrum in vivo imaging system (IVIS) (Perkin Elmer, Norwalk, CT, USA). After five weeks of tests, mice in the orthotopic implantation groups were sacrificed after anesthesia using 1% pentobarbital hydrate. The mice that did not breathe were scored as dead and were dissected. Lungs were excised, observed by the naked eye, fixed, sectioned, and subjected to hematoxylin and eosin (H&E) staining. Histological analyses and lung nodules in serial sections were quantified under a Zeiss microscope axio scope A1 (Zeiss, Oberkochen, Germany).

### Transwell assay

The upper chamber of a Transwell apparatus (Corning Inc., Corning, NY, USA) was precoated with 50 μL of Matrigel solution. MDA-MB-468 cells (2 × 10^5^) were starved overnight and seeded into the upper chamber in serum-free medium, and medium supplemented with 20% FBS was added to the bottom chamber. After 24 h incubation, the cells were fixed with 4% paraformaldehyde and dyed with crystal violet. Typical images of invading cells were obtained for statistical analyses. Each group was imaged at least three fields and three independent experiments. The migration assay was performed in a similar manner to the invasion assay but without the Matrigel pre-coating. Cells were imaged using DIC model of Lecia DMI8 microscope with a 10× objective lens under constant exposure (100 ms) and gain (1.0).

### 3D tumor spheroid invasion assays

MDA-MB-468 cells (1 × 10^4^) in all groups were starved overnight and resuspended in 10 μL of medium containing 5 μL of Matrigel. 1 μL of the cells/collagen mixture were seeded into a Glass Bottom Cell Culture Dish (NEST SCIENTIFIC INC. Woodbridge, NJ, USA) to form spheroids, and were then maintained in a 37 °C incubator supplied with 5% CO_2_ for 1 h. Thereafter, 50 μL of medium containing 20 μL of Matrigel and 10 μL of FBS were quickly coated in the dish subface and maintained in a 37 °C incubator supplied with 5% CO_2_ for 2 h. 1 mL Dulbecco’s Modified Eagle’s Medium (Gibco) containing 10% FBS were added to the dishes and maintained in a 37 °C incubator supplied with 5% CO_2_ for 48 h. Cells were imaged using Lecia DMI8 microscope under a 4× objective lens 50ms-exposure and 1.0-gain.

### CpstFRET analysis

The effectiveness of FRET in the stable monoclonal cell line was determined by the dipole angle between the donor (eCFP) and acceptor (eYFP). Thereafter, the FRET analysis was performed as previously reported [31]. The images at the donor emission and acceptor emission were recorded through a Dual View 2 splitter (MAG biosystems at BioVision Technologies, Exton, PA, USA). The donor and FRET emissions were at 436 nm, the acceptor emission was collected in the donor emission region (455–485 nm), and the FRET emission was collected in the acceptor emission region (520–550 nm). Cells were imaged with a 63× objective lens under the constant exposure (600 ms) and gain (2.0). For the signal cell FRET analysis, FRET/Acceptor emission ratios were calculated for each pixel clearest optical plane for each image field. The cell was first selected by generating a binary mask using the drawing tool in Image J. A Donor mask was generated by applying a threshold on the Donor image, which was then made binary by converting pixel intensity values greater than the Donor threshold to 1 and those lower than it to 0. A similar mask was generated for the acceptor channel. Ratio images (32-bit) were calculated the CFP/FRET ratio (the intensity of the CFP channel divided by the intensity of the FRET channel) using the equation E = eCFP_donor_/eYFP_acceptor_, which correlated negatively correlated with FRET efficiency, but positively with force. For presentation purposes, pseudocolour was applied using ImageJ software in order to obtain the final images.

In the Fluorescence Recovery after Photobleaching (FRAP) test, we selected a region of interest (ROI) and bleached it with a 590 nm laser at 100%. Time-series images were acquired before and after bleaching in 200 s, then we recorded the fluorescence intensity in the ROI and calculated the fluorescence recovery rates. The fluorescence intensities obtained were normalized to the average pre-bleach values in the GraphPad Prism software. In an acceptor bleaching FRET test (FRET-AB), we bleached the acceptor with a 514 nm laser at 100% 20 times, and then the fluorescence intensity of the donor and acceptor after bleaching was recorded. We calculated the FRET efficiency using the equation E = (E_donor-after_-E_donor-before_)/E_donor-before_. Cells were imaged using the FRAP or FRET-SE model of Lecia SP8 confocal microscopy with a 63× objective lens under the constant exposure (600 ms) and gain (2.0).

### Live cell imaging and quantification of Clip170

Cells expressing Clip170 or mutation probes were grown under humidified air containing 5% CO_2_ in Dulbecco’s Modified Eagle’s Medium or Leibovitz L-15 Medium. The Live cell imaging was done in ambient room gas in HEPES buffer solution (100 nM HEPES, 100 nM NaCl, 10 mM Na_2_HPO_4_, PH = 7.4). Because of the strong autofluorescence from serum-containing medium, the culture media was substituted with 37 °C prewarmed HEPES buffer solution, for all FRET and live fluorescence imaging, as described by our previous study [32].

Analysis of the comet lengths, comet quantities, and fluorescence intensities in Figs. 2, 4 and 7 were performed in ImageJ. Firstly, the images were translated into 32-bit depth images and the parameter of Clip170 was analyzed after subtraction of external background via Threshold function in ImageJ. About 70 Clip170 comets were selected randomly in 6 cells for each group and calculated length, quantity and fluorescence intensity via Analysis-Measure function in ImageJ. Statistical analysis of these data was performed in SPSS 22.0 and statistical graphs were performed in Graphpad Prism 7.

### Bioinformatic analyses and clinical samples

Fresh breast cancer tissue samples and adjacent normal tissues were obtained from the Xuzhou Central Hospital (Xuzhou, China). Informed consent was obtained from all patients and ethical approval for this study was obtained from the Institutional Review Board of Human Research of the Xuzhou Central Hospital.

The survival analysis data were obtained from the Kaplan–Meier plotter database (http://kmplot.com/analysis/) [33]. All patients were sorted into different groups, and the data were tested for significance using a log-rank test.

### Long-time imaging analysis

The MDA-MB-468 cells were cultured under humidified air containing 5% CO_2_ at 37 °C. Cells were cultured in a 12-well plate until they reached 50% confluence. After that, cells were cultured in Dulbecco’s Modified Eagle’s Medium without fetal bovine serum during Long-time imaging. The 12-well plates were imaged every 30 min for 8 h in the IncuCyte Zoom time-lapse microscopy system (Essen, USA), equipped with an IncuCyte Zoom, 10×objective lens. Each group was imaged six fields and three independent experiments.

### Plate cloning formation experiment

MDA-MB-468 cells (2 × 10^3^) in all groups were digested and inoculated into a culture dish. Culture medium was changed every 2 days and the assay was performed for 2 weeks. After washing with PBS, 4% Paraformaldehyde solution was used to fix cells for 30 min at room temperature. Then, 500 μL of crystal violet solution was used to fix the cells for 30 min at room temperature. After washing and air drying, clones were counted using a cloning counter.

### Statistical analysis

Data are shown as the mean ± SEM and were analyzed using Student’s t test. One-way analysis of variance (ANOVA) was conducted using SPSS v.22.0 (IBM Corp.Armonk, NY, USA) for single-factor sample comparisons. Tukey’s post hoc test was used for other comparisons between two means. Each experiment was repeated at least three times. More than six cells were imaged, and each condition was analyzed independently.

## Results

### Higher Clip170 tension correlates with breast cancer aggressiveness

**Fig. 1:**
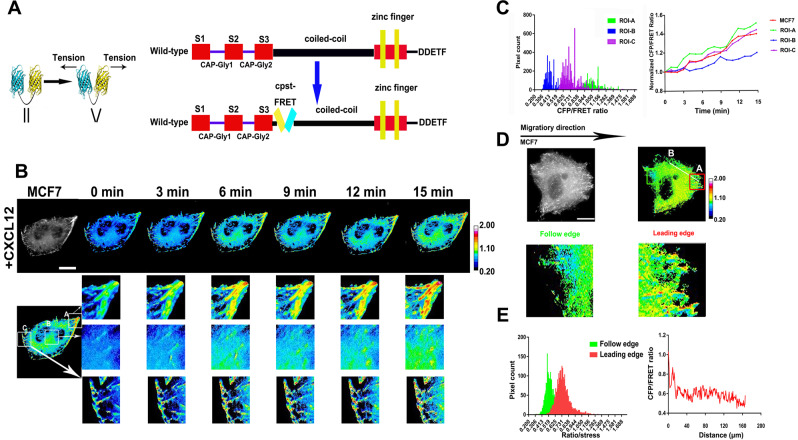
Positive correlation between Clip170 tension and breast cancer invasion and metastasis. **A** In the mTurquoise2–7aa-sYFP2 (cpstFRET), mTurquoise2 (cyan) is the donor and sYFP2 (yellow) is the acceptor, and they form an angle with external tension while they are parallel in the absence of external tension. As the external force increases, the fluorescent resonance energy transfer (FRET) efficiency of the probes are reduced by broadening of the angle of cpstFRET. The construction of the Clip170 plasmid and Clip170 probe are shown. The Clip170 probe with cpstFRET is inserted between amino acid 344 and 345 of Clip170. **B** 15-min time-lapse images of MCF7 cells after CXCL12 treatment. Calibration bar, 0.2–2.0; scale bar, 10 μm. **C** Left panel: Pixel count distribution analysis of CFP/FRET ratios in different ROI of MCF7 cells. Right panel: Normalized CFP/FRET signals in whole cell corresponding to tension versus time. **D** Representative CFP and CFP/FRET images of directional migratory MCF7 cells are presented. **E** Left panel: The pixel count distribution of CFP/FRET ratios in leading edge and follow edge. Right panel: The pixel count distribution of CFP/FRET ratios in leading edge and cell periphery. Calibration bar, 0.2–2.0; scale bar, 10 μm.

Role of the MF force on cancer invasion and migration has been widely clarified [5]. However, how the MT force and its transmission regulate cancer invasion and migration are still unclear. In the present study, MT force transmission in breast cancer cell aggressiveness was explored, which might be associated closely with plus-end tracking proteins Clip170. Based on the previous reports [31, 32, 34, 35], we designed a cpstFRET tension probe between Clip170 Serine-rich region 3 and coiled-coil domain to detect real-time tension variation in Clip170 (Fig. 1A). After that, FRET-AB and FRAP analysis were performed to test cpstFRET-Clip170 probe validity. In the FRET-AB test, the transient FRET efficiency of the probe was determined as 13.26% (Supplementary Fig. 1A). Meanwhile, in the FRAP test, the recovery rate for Clip170 in the ROI was determined as 71.8% after 250s-bleaching (Supplementary Fig. 1B). Based on our previous study, the MT tension decreased after dynein inhibitor ciliobrevin D (Cil D) and microtubule depolymerizing agent treatment, respectively [34]. The Clip170 tension indicated a sensitive variation while the MT tension decreased (Supplementary Fig. 1D). The data reveal that MT force contributes major power for Clip170 tension and the cpstFRET-Clip170 probe is sufficiently effective and sensitive for real-time monitoring of intracellular structure tension.

Furthermore, to identify dynamic Clip170 tension variation during breast cancer migration and invasion, we transfected the cpstFRET-Clip170 probe into MCF7 and MDA-MB-231 cells, respectively, and established a short-term aggression model induced by CXCL12 stimuli. As shown in Fig. 1 and Supplementary Fig. 1, Clip170 tension revealed an increasing trend after 15-min CXCL12 stimuli both in MCF7 (Fig. 1B) and MDA-MB-231 cells (Supplementary Fig. 1E). Subsequently, pixel count distribution analysis of Clip170 tension in different subcellular locations indicated that Clip170 tension at the cell edge (ROI-A) was higher than those at middle parts (ROI-B and C). Clip170 tension at the cell edge (ROI-A) also revealed a faster increasing trend after CXCL12 stimuli than those at the middle parts (ROI-B and C) (Fig. 1C and Supplementary Fig. 1F).

To further investigate the mechanical mechanism of Clip170 in breast cancer invasion and migration, we established a directional migration model through matrigel with a serum concentration gradient. MCF7, SKBR3, MDA-MB-231, and MDA-MB-468 cells transfected with cpstFRET-Clip170 could migrate along the serum concentration [36, 37]. In directional migratory MCF7 cells, pixel count distribution analysis suggested that Clip170 tension at the leading edge was high, while those in the follow edge and cell periphery were relatively low (Fig. 1D, E). In summary, these results indicate that Clip170 tension is positively related to aggressive motility both in the short-term aggressive model and the directional migration model. In addition, the subcellular location of Clip170 tension is correlated with the migration direction.

### α-tubulin detyrosination-induced MT structure disassembly is correlated with Clip170 tension and subcellular phenotype during breast cancer invasion and migration

**Fig. 2:**
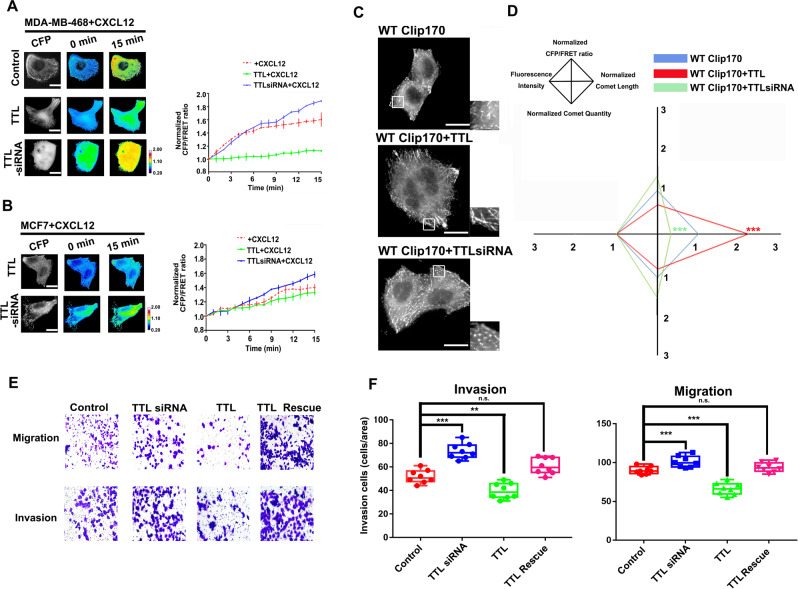
TTL expression regulates Clip170 tension and breast cancer invasion and migration. **A** Left panel: 15-min time-lapse images of FRET analysis in MDA-MB-468 cells expressing the Clip170-cpstFRET probe and transfected with the *TTL* siRNA or *TTL* overexpression plasmid after CXCL12 treatment. Calibration bar, 0.2–2.0; scale bar, 10 μm. Right panel: Normalized CFP/FRET signals for the whole cell corresponding to tension versus time, respectively. (Mean ± SEM, *n* ≥ 6 cells in each group) (**B**) Left panel: 15-min time-lapse images of FRET analysis in MCF7 cells expressing the Clip170-cpstFRET probe and transfected with the *TTL* siRNA or *TTL* overexpression plasmid after CXCL12 treatment. Calibration bar, 0.2–2.0; scale bar, 10 μm. Right panel: Normalized CFP/FRET signals for the whole cell corresponding to tension versus time, respectively. (Mean ± SEM, *n* ≥ 6 cells in each group). **C** Representative CFP images of MDA-MB-468 cells expressing the Clip170-cpstFRET probe and transfected with the *TTL* siRNA or *TTL* overexpression plasmid. **D** Normalized CFP/FRET signals, comet lengths, comet quantities, and fluorescence intensities for the whole cell in different groups (scale bar, 10 μm, *n* ≥ 6 cells in each group, ***P < 0.001 compared with WT-Clip170 alone group). **E** Representative images of the migration and invasion of MDA-MB-468 cells in the control, *TTL* siRNA, *TTL* overexpression and *TTL* rescue groups. **F** Quantitative analysis of the migration and invasion cells (Mean ± SEM, n = 3 independent experiments and at least three fields, ***P < 0.001 compared with WT-Clip170 alone group).

Role of MT force and its transmission depends on cytoskeleton alteration, which is associated with the tubulin tyrosination level [38]. TTL specifically accelerates α-tubulin tyrosination to influence the interaction with +TIPs, including Clip170 [21, 39]. To analyze the MT and Clip170 mechanical properties after MT structural alteration, MCF7 cells were co-transfected with a *TTL* siRNA or *TTL* overexpression plasmid and the cpstFRET-tubulin probe. The α-tubulin tyrosination/detyrosination caused by TTL protein level was confirmed by western blotting (Supplementary Fig. 2A). Compared with control group, *TTL* overexpression inhibited the MT force increment after 15-min CXCL12 stimuli, especially at the cell edges. The *TTL*-siRNA reversed this tendency (Supplementary Fig. 2B-D). After that, MDA-MB-468 cells were co-transfected with a *TTL* siRNA or *TTL* overexpression plasmid and the cpstFRET-Clip170 probe. Clip170 tension was decreased in the *TTL* overexpression group after 15-min CXCL12 treatment. Meanwhile, *TTL* knockdown (KD) resulted in a more significant upward tendency of Clip170 tension (Fig. 2A). Similar results were shown in MCF7 (Fig. 2B), SKBR3 (Supplementary Fig. 2E), and MDA-MB-231 cells (Supplementary Fig. 2F). These results reveal that MT alteration induced by α-tubulin tyrosination could reduce Clip170 tension during the aggressive behavior of breast cancer.

**Fig. 3:**
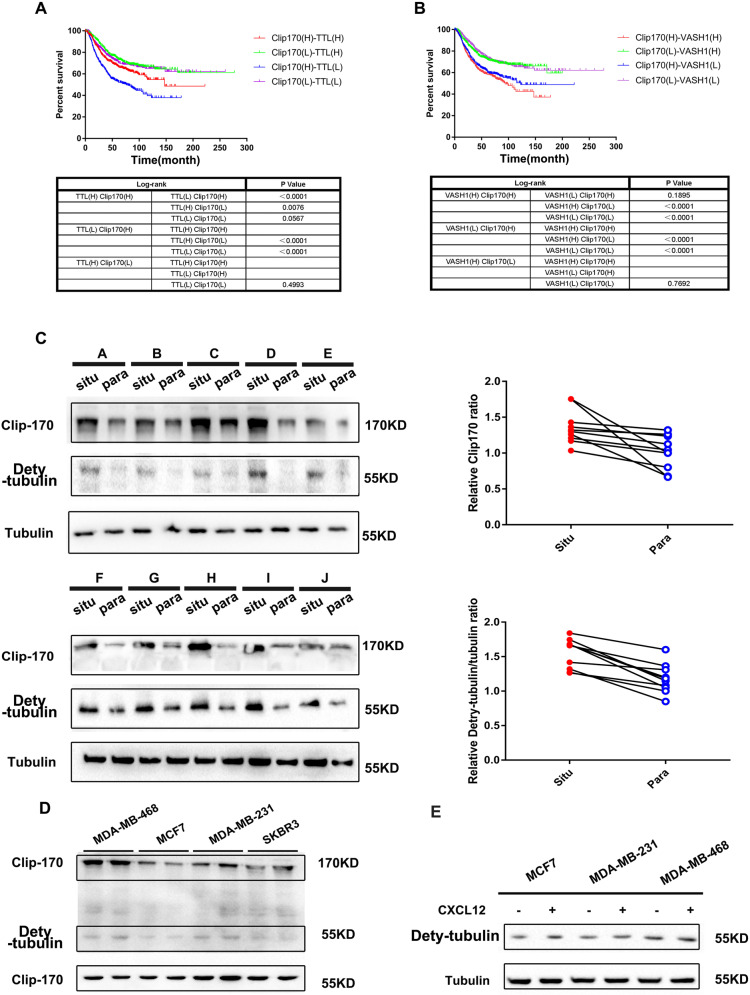
Association between Clip170 and α-tubulin detyrosination levels with prognosis and aggressiveness in breast cancer. **A** Kaplan–Meier plots of the overall survival of patients with breast cancer, stratified by Clip170 and TTL expression. **B** Kaplan–Meier plots of the overall survival of patients with breast cancer, stratified by Clip170 and VASH1 expression. **C** Left panel: Western blotting analysis of Clip170 and detyrosinated-tubulin levels in fresh breast cancer tissue (situ) and adjacent normal tissue (para). Right panel: Quantitative analysis of Clip170 and detyrosinated-tubulin levels in breast cancer tissues and matched normal tissues (*n* = 10). **D** Western blotting analysis of Clip170 and detyrosinated-tubulin levels in MDA-MB-231, MDA-MB-468, MCF7, and SKBR3 cells. **E** Western blotting analysis of detyrosinated-tubulin levels in MDA-MB-231, MDA-MB-468, and MCF7 cells after CXCL12 treatment.

Previous studies suggest an intimate connection between Clip170’s phenotype and its activity [17]. To investigate whether TTL could influence Clip170 tension via its phenotype, we quantified the length and quantity of Clip170 comets. In contrast to the discrete comet-like Clip170 distribution in the control group, the *TTL* KD cells exhibited significantly more but shorter comets, which displayed a higher Clip170 tension and a fraction of Clip170 mislocalization in the cytoplasm. *TTL* overexpressing cells showed less but longer comets than those in the control group (Fig. 3C). Furthermore, we investigated whether the α-tubulin tyrosination level could regulate breast cancer progression via Clip170. Flow cytometry analysis and plate cloning formation experiment showed that TTL had no significant effect on apoptosis and proliferation in MDA-MB-468 cells (Supplementary Fig. 2G, H). 3D spheroid invasion assays suggested that α-tubulin tyrosination correlated negatively with breast cancer aggression (Supplementary Fig. 2I). In addition, Transwell assays also revealed that *TTL* overexpressing cells displayed attenuated aggressiveness and *TTL* KD cells showed intensified invasion and migration abilities (Fig. 2E, F). To summarize, α-tubulin tyrosination induced by TTL could reduce Clip170 tension, accompanied with fewer but longer Clip170 comets, which then restrains breast cancer cell aggressiveness.

### α-tubulin detyrosination and Clip170 are positive associated with malignancy and poor prognosis in breast cancer

To analyze the role of Clip170 and α-tubulin tyrosination in patient prognosis, Kaplan–Meier survival analysis was used to verify the association between levels of Clip170, TTL, and the prognosis of patients with breast cancer. Patients with higher Clip170 and lower TTL expression level had a shorter overall survival (Fig. 3A). Based on previous studies, VASH1 specifically recognized and catalyzed α-tubulin detyrosination [40, 41]. Similarly, patients with higher Clip170 and VASH1 coexpression had a poorer prognosis (Fig. 3B). Next, we analyzed the detyrosinated-tubulin and Clip170 levels of 10 patients with breast cancer using western blotting. The results showed higher detyrosinated-tubulin and Clip170 levels in cancer tissues than those in paired normal breast tissues (Fig. 3C). Thereafter, the levels of detyrosinated-tubulin and Clip170 in four different breast cancer cell lines were detected. Compared with SKBR3 and MCF7 cells, MDA-MB-231 and MDA-MB-468 cells suggested a higher degree of malignancy according to their higher levels of detyrosinated-tubulin (Fig. 3D). Meanwhile, the detyrosinated-tubulin levels in MCF7, MDA-MB-468, and MDA-MB-231 increased slightly after CXCL12 treatment (Fig. 3E). These data indicate a positive correlation between the α-tubulin detyrosination levels and Clip170 with malignancy and poor prognosis in breast cancer.

### Clip170 phosphorylation is correlated with Clip170 tension and subcellular phenotype during breast cancer invasion and migration in vivo and in vitro

**Fig. 4:**
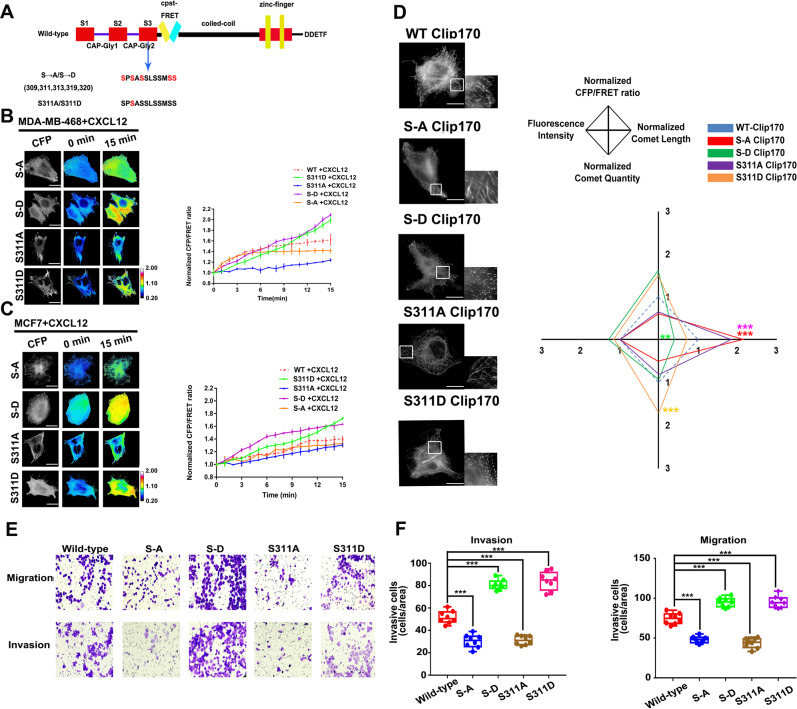
Clip170 phosphorylation increases Clip170 tension and breast cancer invasion and migration. **A** The construction of the Clip170 mutation probes. **B** Left panel: 15-min time-lapse images MDA-MB-468 cells transfected with Clip170-S-D-cpstFRET, Clip170-S-A-cpstFRET, Clip170-S311D-cpstFRET or Clip170-S311A-cpstFRET probes after CXCL12 treatment. Calibration bar, 0.2–2.0; scale bar, 10 μm. Right panel: Normalized CFP/FRET signals for the whole cell corresponding to Clip170 tension versus time (Mean ± SEM, *n* ≥ 6 cells in each group). **C** Left panel: 15-min time-lapse images MCF7 cells transfected with Clip170-S-D-cpstFRET, Clip170-S-A-cpstFRET, Clip170-S311D-cpstFRET or Clip170-S311A-cpstFRET probes after CXCL12 treatment. Calibration bar, 0.2–2.0; scale bar, 10 μm. Right panel: Normalized CFP/FRET signals corresponding to Clip170 tension versus time (Mean ± SEM*, n* ≥ 6 cells). **D** Left panel: Representative images of MDA-MB-468 expressing Clip170-cpstFRET, Clip170-S311D-cpstFRET, Clip170-S311A-cpstFRET, Clip170-S-D-cpstFRET, and Clip170-S-A-cpstFRET probes. Right panel: Normalized CFP/FRET signals, comet lengths, comet quantities, and fluorescence intensities for the whole cell in the different groups (scale bar, 10 μm, *n* ≥ 6 cells in each group, **P < 0.01, ***P < 0.001 compared with WT-Clip170 group). **E** Representative images of the migration and invasion of MDA-MB-468 cells in the WT, S311A, S311D, S-A, and S-D groups. **F** Quantitative analysis of the migration and invasion rates (Mean ± SEM, n = 3 independent experiments and at least three field, ***P < 0.001 compared with WT-Clip170 group).

Clip170 activity could be closely associated with its tension. A previous study suggests that the third serine-rich region of Clip170 could influence Clip170 activity via phosphorylation. Clip170 can be phosphorylated on Ser309, Ser311, Ser313, Ser319, and Ser320 at the third serine-rich region [42]. Consequently, we constructed and tested two phosphomimetic mutation Clip170 FRET probes (Clip170-S311D-cpstFRET and Clip170-S-D-cpstFRET) and two phosphorylation-deficient mutation Clip170 FRET probes (Clip170-S311A-cpstFRET and Clip170-S-A-cpstFRET) (Fig. 4A). Both Clip170-S-A and Clip170-S311A inhibited the increase of Clip170 tension after CXCL12 treatment in MDA-MB-468 cells. Meanwhile, the increased Clip170 tension caused by CXCL12 could be intensified by the Clip170-S-D and Clip170-S311D mutations (Fig. 4B). Similar results were shown in MCF7 cells (Fig. 4C). These results indicate that Clip170 phosphorylation-induced activity variation could enhance its tension transmission.

To investigate whether the Clip170 mutations influenced Clip170 tension through its phenotype, we quantified the length and quantity of Clip170 comets. Compared with the wild-type (WT) group, phosphomimetic mutation shortened the length of comets and increased its quantity. Most Clip170 showed a spotty pattern distribution in the S311D and S-D mutation groups, and the phosphorylation-deficient mutated Clip170 exhibited significantly fewer but longer comet-like distribution (Fig. 4D). As mention above, MDA-MB-468 cells were transfected under the same condition in WT and mutation groups and images under the same exposure and gain. Cells in the WT-Clip170 groups revealed similar phenotype with the cells without transfection and treatment. After that, we detected the CFP expression levels and the results showed that the ectogenic cpstFRET-Clip170 probe and Clip170 mutated probes have been controlled at the same level in different groups (Supplementary Fig. 3A). These data indicates that the phenotype differences between the Clip170 mutated groups were not caused by the different level of overexpression. Furthermore, we investigated whether Clip170 mutation could regulate breast cancer progression. Flow cytometry analysis and plate cloning formation experiment showed that Clip170 phosphorylation level had no effect on MDA-MB-468 cell proliferation and apoptosis (Supplementary Fig. 3B, C). 3D spheroid invasion assays suggested that Clip170 phosphomimetic mutation can promote the infiltration of breast cancer (Supplementary Fig. 3D). Transwell assays also revealed that MDA-MB-468 cells with the phosphorylation-deficient mutation of Clip170 displayed attenuated aggressiveness, while cells in the S311D and S-D mutation group showed intensified invasion and migration abilities (Fig. 4E and F). Therefore, we suggest that breast cancer progression could be enhanced by increasing the Clip170 phosphorylation level, which changes its phenotype and force.

**Fig. 5:**
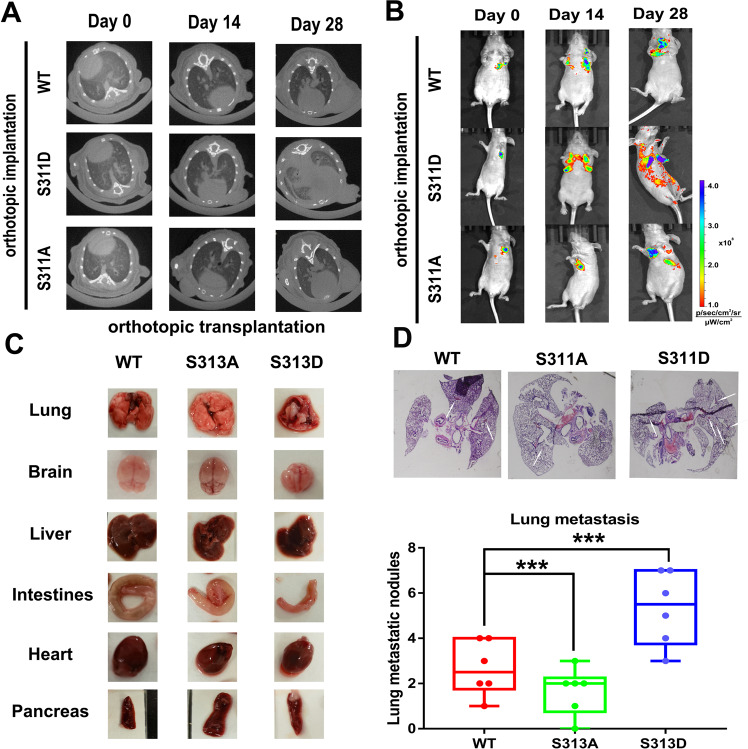
Clip170 phosphorylation promotes breast cancer invasion and migration in vivo. **A** For the orthotopic implantation assays, bioluminescent images of systemic metastases in nude mice with Clip170-WT, Clip170-S311A, and Clip170-S311D MDA-MB-468 cells are shown. Bioluminescence calibration bar, 1.0 × 10^8^–1.0 × 10^9^. **B** Representative images of the micro CT analysis of lungs from nude mice in the orthotopic implantation assay. **C** Representative images of lung, brain, liver, intestine, heart, and pancreas tissues from nude mice in the orthotopic implantation assay. **D** Representative images of H&E-stained histological sections of lung from nude mice in the orthotopic implantation assay (white arrows: metastatic nodules, *n* = 6, ***P < 0.001 compared with WT group).

Furthermore, to confirm the effect of Clip170 phosphorylation on breast cancer aggressiveness in vivo, Clip170-cpstFRET, Clip170-S311A-cpstFRET, and Clip170-S311D-cpstFRET stable MDA-MB-468 cell lines were constructed. These cells were transplanted into BALB/c nude mice fat pads for orthotopic implantation assays or injected in the tail vein. IVIS spectra showed that mice in Clip170-S311D group developed more spontaneous metastatic signals compared with mice in the WT group. Meanwhile, the Clip170-S311A group revealed weaker fluorescence intensity in both the orthotopic implantation groups (Fig. 5B) and the tail vein injection groups (Supplementary Fig. 3E). Furthermore, the micro-CT assay suggested that the Clip170-S311D group showed a marked pulmonary shadow and the number of distinct metastatic sites in the Clip170-S311D group was significantly higher than those in the Clip170-WT and Clip170-S311A group (Fig. 5A and Supplementary Fig. 3F), as demonstrated by lung anatomy and H&E staining (Fig. 5C, D). These results indicate that the level of Clip170 phosphorylation regulates breast cancer aggressiveness in vivo, and is associated with Clip170 tension.

EMT is implicated in carcinogenesis and confers metastatic properties on cancer cells. During EMT, epithelial cells lose their polarized organization and acquire migratory and invasive properties [43, 44]. We explored whether Clip170 phosphorylation could influence EMT. Long-time imaging showed that compared with the cells in WT group, cells in phosphomimetic mutation groups revealed a higher length-to-width ratio after 8 h-TGFβ treatment (Supplementary Fig. 4A, B). We then analyzed the levels of EMT biomarkers E-cadherin, N-cadherin, and vimentin, using western blotting. N-cadherin and vimentin levels in the phosphomimetic mutation group were higher than those in the WT group after TGFβ treatment (Supplementary Fig. 4C). These results suggested that cells with Clip170 phosphomimetic mutation were more likely to generate EMT after TGFβ stimuli.

### RSK increases Clip170 tension and breast cancer aggressiveness by inducing Clip170 phosphorylation

**Fig. 6:**
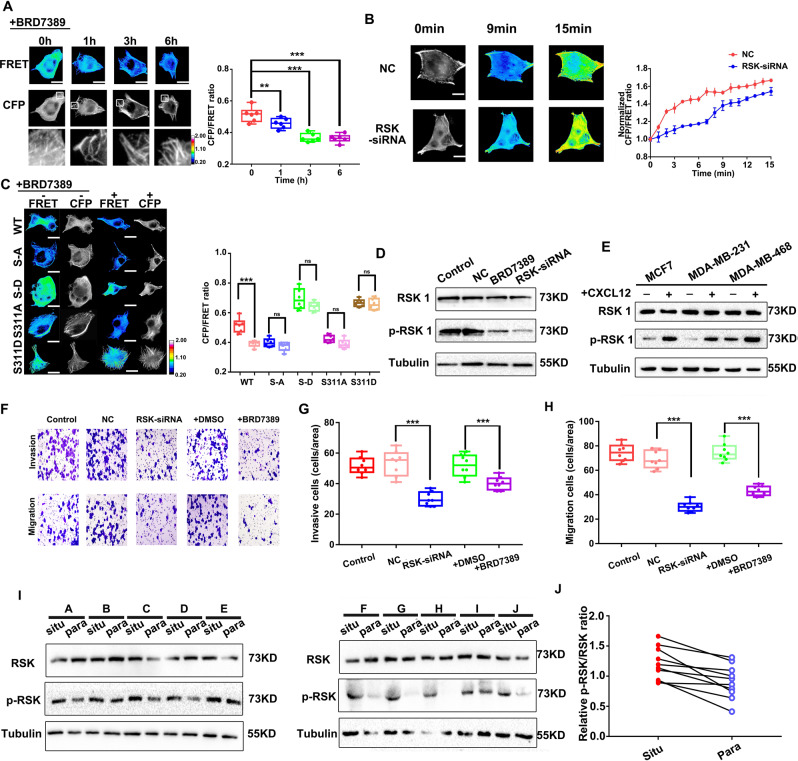
RSK promotes Clip170 tension and breast cancer aggressiveness. **A** Left panel: Representative images of FRET analysis of cells treated with the RSK inhibitor (BRD7389) for 0, 1, 3, and 6 h. Calibration bar, 0.2–2.0; scale bar, 10 μm. Right panel: Mean CFP/FRET ratios for the whole cell. (mean ± SEM, *n* ≥ 6 cells in each group, **P < 0.01, ***P < 0.001 compared with 0h-treatment group). **B** Left panel: 15-min time-lapse images of FRET analysis in MDA-MB-468 cells transfected with the Clip170-cpstFRET probe alone or combined with *RSK* siRNA. Calibration bar, 0.2–2.0; scale bar, 10 μm. Right panel: Normalized CFP/FRET signals for the whole cell corresponding to Clip170 tension versus time (mean ± SEM, *n* ≥ 5 cells in each group). **C** Left panel: Representative images of FRET analysis of MDA-MB-468 cells transfected with the Clip170-cpstFRET, Clip170-S-A-cpstFRET, Clip170-S-D-cpstFRET, Clip170-S311A-cpstFRET, or Clip170-S311D-cpstFRET probes treated with the RSK inhibitor (BRD7389) for 6 h. Calibration bar, 0.2–2.0; scale bar, 10 μm. Right panel: Mean CFP/FRET ratios for the whole cell. (mean ± SEM, *n* ≥ 6 cells in each group, ***P < 0.001 compared with the cells without BRD7389 treatment). **D** Western blotting analysis of *RSK*-siRNA and RSK inhibitor treatment in MDA-MB-468 cells. **E** Western blotting analysis of RSK and p-RSK levels in MDA-MB-468, MCF7, and MDA-MB-231 after CXCL12 treatment. **F** Representative images of the migration and invasion of control, NC, *RSK*-siRNA, DMSO, and RSK inhibitor (BRD7389) treatment groups. **G–H** Quantitative analysis of the migration (**G**) and invasion (**H**) rates (Mean ± SEM, *n* = 3 independent experiments and at least three fields, ***P < 0.001 compared with NC or DMSO group). **I** Western blotting analysis of RSK and p-RSK levels in breast cancer tissue (situ) and adjacent normal tissue (para). **J** Quantitative analysis of the p-RSK/RSK ratio in breast cancer tissue and matched normal tissues (*n* = 10).

Based on previous studies, RSK (Ribosomal protein S6 kinase) activation promotes breast cancer cell motility and migration [45, 46]. Meanwhile, RSK is also speculated to act as a regulator of Clip170 via S311 phosphorylation [42]. To investigate whether RSK could regulate Clip170 tension via S311 phosphorylation, the RSK inhibitor BRD7389 was used to treat Clip170-cpstFRET stable MDA-MB-231 cell lines. Clip170 tension showed a clear downward trend after BRD7389 treatment, and the comet length of Clip170 increased obviously (Fig. 6A). We transfected Clip170-cpstFRET stable MDA-MB-468 cells with a *RSK*-siRNA or NC, and then treated the cells with CXCL12 for 15 min. Compared with cells in the NC group, the Clip170 tension of cells in the *RSK*-siRNA group was maintained at a lower level (Fig. 6B). Meanwhile, RSK activity was elevated after CXCL12 treatment in MDA-MB-231, MDA-MB-468, and MCF7 cells (Fig. 6E). However, tension in Clip170 phosphomimetic mutation groups and phosphorylation-deficient mutation group showed no obvious variation after 6h-BRD7389 treatment (Fig. 6C, D). These results indicate that RSK regulates Clip170 tension via S311 phosphorylation. After that, Transwell assays revealed that RSK inhibition and knockdown could decrease breast cancer invasion and migration (Fig. 6F). Furthermore, we analyzed the level of RSK and phosphorylated (p)-RSK in samples from 10 patients with breast cancer using western blotting. These results showed that the p-RSK level was elevated in breast cancer tissue in situ compared with paired para-carcinoma tissue (Fig. 6G). In conclusion, our results suggest that RSK inhibition reduces breast cancer aggressiveness by decreasing Clip170 tension and increasing the length of comets by regulating the Clip170 phosphorylation level.

### IQGAP1 subcellular localization is correlated with Clip170 tension transmission to promote the formation of cortactin-associated filopodia and lamellipodia and breast cancer metastasis

**Fig. 7:**
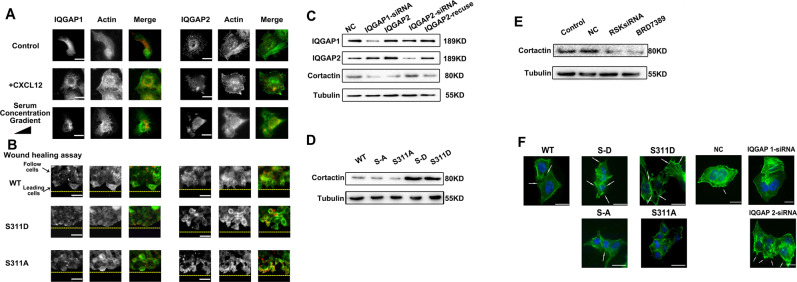
IQGAP 1 location regulates cortactin-associated formation of filopodia and lamellipodia. **A** Representative images of MF and IQGAP1 or IQGAP2 distribution in the directional migration model or after CXCL12 treatment. (green, FITC-stained MFs; red, TRITC-stained IQGAP1/IQGAP2), scale bar, 10 μm. **B** Representative images of MF and IQGAP1 or IQGAP2 distribution in the wound healing assay. (green, FITC-stained MFs; red, TRITC-stained IQGAP1/IQGAP2), scale bar, 10 μm. **C** Western blotting analysis of Cortactin, IQGAP1, and IQGAP2 levels in the NC, *IQGAP1*-siRNA, *IQGAP*2-siRNA, and *IQGAP2* and *IQGAP2* rescue groups. **D** Western blotting analysis of cortactin levels in the WT, S311A, S311D, S-A, and S-D groups. **E** Western blotting analysis of cortactin levels in the control, NC, *RSK-* siRNA, and RSK inhibitor treatment groups. **F** Representative images of filopodia and lamellipodia structures in the Clip170 mutation, and NC, *IQGAP1* and *IQGAP2*-siRNA groups (scale bar, 10 μm, green, FITC-stained MFs; blue, nucleus; white arrows: filopodia and lamellipodia structures).

Breast cancer cell aggressiveness relies on the formation of filopodia and lamellipodia [47], and the role of MT-associated Clip170 tension in this process is unclear. IQGAP1 and 2 are considered to be scaffolding proteins that regulate MT dynamics and assembly of the MF cytoskeleton by combining with Clip170 [48, 49]. We hypothesized that the IQGAP family might play an important role in Clip170 tension to form filopodia and lamellipodia. Immunofluorescence assays showed that IQGAP1 and IQGAP2 were mainly concentrated in the cytoplasm before CXCL12 treatment. However, in the directional migratory cells, IQGAP1 distributed along serum concentration and localized at the leading edges, and IQGAP2 remained concentrated in the cytoplasm (Fig. 7A). In the wound healing assay, IQGAP1 location was influenced by the level of Clip170 phosphorylation. IQAGP1 was mainly localized to the leading edges of cells in the S311D group, whereas in the S311A group, IQGAP1 was widely distributed in the cytoplasm of cells. Meanwhile, IQGAP2 was widely distributed in the cytoplasm in all groups (Fig. 7B). These data suggest that breast cancer aggressiveness variation induced by Clip170 phosphorylation mainly affects IQGAP1 location rather than IQGAP2.

Filopodia and lamellipodia formation are considered as highly aggressive phenotypes in breast cancer, and whether IQGAP1 and IQGAP2 are correlated with the formation of filopodia and lamellipodia was unclear. The knockdown of *IQGAP1* and *IQGAP2* was confirmed by western blotting (Fig. 7C). Western blotting analysis suggested that *IQGAP2* knockdown upregulated the level of the cortical actin marker cortactin, and *IQGAP1* knockdown showed the opposite phenotype (Fig. 7C). Immunofluorescence analysis showed that cells in the *IQGAP2*-siRNA group generated more filopodia and lamellipodia, and cells in the *IQGAP1*-siRNA group showed decreased numbers of filopodia and lamellipodia (Fig. 7F). Accordingly, compared with cells in the WT group, the Clip170 phosphomimetic mutation upregulated the cortactin expression level, and increased the numbers of filopodia and lamellipodia. Clip170 phosphorylation-deficient mutation resulted in fewer filopodia and lamellipodia, accompanying by cortactin downregulation (Fig. 7D, F). RSK inhibition also reduced cortactin level (Fig. 7E). These results suggested that Clip170 tension transmitted to the cell periphery is associated with IQGAP1 location, accompanied by upregulation of cortactin levels to promote the formation of filopodia and lamellipodia.

**Fig. 8:**
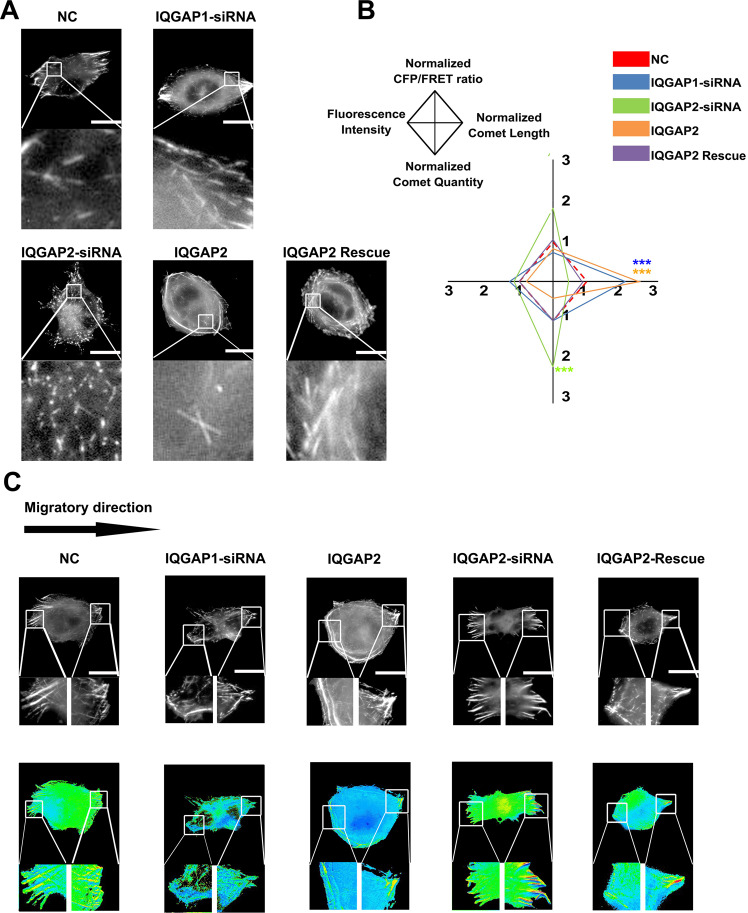
IQGAP1 and 2 are correlated in Clip170 tension magnitude and distribution. **A** Representative images of MDA-MB-468 expressing the Clip170-cpstFRET probe in the *IQGAP1*-siRNA, *IQGAP2*-siRNA, *IQGAP2* overexpression, and *IQGAP2* rescue groups. **B** Normalized CFP/FRET signals, comet lengths, comet quantities, and fluorescence intensities for the whole cell in the different groups (scale bar, 10 μm, *n* ≥ 6 cells in each group, ***P < 0.001 compared with NC group). **C** Representative images of MDA-MB-468 cells expressing the Clip170-cpstFRET probe in the *IQGAP1*-siRNA, *IQGAP2*-siRNA, *IQGAP2* overexpression, and *IQGAP2* rescue groups in the directional migration model, scale bar, 10 μm.

To further investigate the role of IQGAP1 and IQGAP2 in Clip170 tension transmission, *IQGAP1*-siRNA and *IQGAP2*-siRNA were co-transfected with the Clip170-cpstFRET probe into MDA-MB-468 and MCF7 cells. Compared with the NC group, *IQGAP2* knockdown enhanced the upward trend of Clip170 tension after CXCL12 treatment, and *IQGAP1* knockdown decreased the upward trend. Moreover, *IQGAP2* overexpression weakened the upward trend of Clip170 tension and *IQGAP2* rescue reversed the weakening effect (Supplementary Fig. 5A, B). Furthermore, we quantified the length and quantity of Clip170 comets in MDA-MB-468 cells. Compared with those in the NC group, *IQGAP2* knockdown shortened the length of the comets and increased their quantity, and cells overexpressing *IQGAP2* exhibited significantly fewer, but longer, comets. Meanwhile, *IQGAP1* knockdown only increased the Clip170 comet length but did not affect the quantity (Fig. 8A, B). In the directional migration model, *IQOGP1* knockdown reversed the Clip170 tension difference between the leading and follow edges, and *IQGAP2* knockdown reinforced the difference (Fig. 8C). Therefore, a Transwell assay was performed, which revealed that MDA-MB-468 cells transfected with *IQGAP2*-siRNA displayed attenuated aggressiveness and cells in *IQGAP2* overexpression and *IQGAP1* knockdown groups showed intensified invasion and migration abilities (Supplementary Fig. 5D). These results suggest that IQGAP1 is correlated with Clip170 tension polarization during breast cancer invasion and migration.

## Discussion

Numerous studies have suggested that metastatic tumors exhibit aberrant MT dynamics and increased MT force [50, 51]. In the present study, we find that the MT plus-end tracking protein Clip170 tension depended on the MT force and the motor molecule, dynein, which is positively related to breast cancer mobility and aggressiveness. TTL-associated MT structural assembly promotes Clip170 tension to a higher level. RSK-regulated Clip170 phosphorylation is also correlated with the formation of aggressive structures and the increase of Clip170 tension. Meanwhile, the polarization of Clip170 tension induced by the subcellular distribution of IQGAP1 is closely associated with the directionality of breast cancer migration. Therefore, we suggest Clip170 tension as a marker for breast cancer malignancy and mobility, and as a potential target for the therapy of metastatic cancer.

Clip170 as a linker between the dynein complex and the MT plus end; solely is not sufficient to generate force. Clip170 tension mainly relies on MT force, based on the binding between its CAP-GLY domain and α-tubulin C-terminal EEY sequence at the MT plus end [14, 52]. Clip170 also receives dynein’s traction via the connection between the Clip170 zinc-finger motif and dynactin subunit p150^Glued^ [53, 54]. MT depolymerization and dynein inhibition analysis also suggest that the MT force and motor molecule dynein play a vital role in Clip170 tension (Supplementary Fig. 1D). In migrating cancer cells, dynein is reported to be activated and shows a faster movement rate [55, 56], thereby inducing an MT force increase, resulting in an upward tendency of Clip170 tension (Fig. 1D, E), which is considered to be a positively correlated with cancer invasion and metastasis [7, 57].

Clip170 tension could not only rely on MT-associated mechanical signal, but also on the MT structural assembly. Previous studies indicate that cancer cell movements required accelerated MT dynamics. Inhibition of MT assembly reduces cancer cell mobility and aggressiveness (77, 78). Traditional MT force-generation models also indicate that polymerizing MTs exerted force upon boundaries in a force- and concentration-dependent manner [58, 59]. Meanwhile, in present study, Clip170 tension polarizes at cell protrusions toward the migration direction (Supplementary Fig. 1G-L), which possibly caused by detyrosinated tubulin translocation at leading edge and reveals a higher assembly rate and MT force (Supplementary Fig. 2B) [60, 61]. Furthermore, tubulin detyrosination-induced MT assembly also promotes Clip170 tension to exhibit a sensitive response to chemokine stimuli (Fig. 2). In short, MT assembly rate is an essential factor on Clip170 tension magnitude.

Clip170 displays a spotty pattern or comet-like distribution in breast cancer cells [62, 63], suggesting different activities and tensions. Recent studies explain these distribution difference-induced activity variations by the ‘phase separation’ theory [64, 65]. Different aggregation states of proteins caused by phase separation could affect various protein functions and biological processes [66–68]. Clip170 comets are also reported to be similar to phase-separated liquid condensates [69]. In the present study, different distributions of Clip170 indicate discrepant force transmission efficiency. Phosphorylation-deficient Clip170 mostly shows an elongated discrete comet-like distribution (Fig. 4D) and a lower force transmission efficiency (Fig. 4B, C), accompanied with lower aggressiveness and malignancy (Fig. 4E, F). The elongated Clip170 comets are also reported to reduce the speed of the comets towards the cell periphery and MT dynamics, which decreases the MT extension rate and cell mobility [17, 70, 71]. A previous study suggests that the interaction between the CAP-GLY domain and zinc-finger motif of Clip170 could result in intermolecular polymerization [15]. Scanning force microscopy also suggests that unphosphorylated Clip170 could form end-to-end oligomerization [72]. Therefore, we speculate that the phosphorylation-deficient Clip170 might form oligomers because of intermolecular end-to-end association, resulting in only a few Clip170 molecules being involved in MT force transmission. Similarly, phosphorylated Clip170 displays a spotty distribution (Fig. 4D) and a faster and higher Clip170 tension variation (Fig. 4). We speculate that phosphorylated Clip170 might be inclined to form synclastic parallel dimers and aggregate into shorter comets [71, 73, 74]. Besides, phosphorylated Clip170 is more likely to accumulate at the more distal MT end in metastatic cancer cells [17], compared with non-phosphorylated Clip170, which experiences a stronger pull by MTs. Therefore, it is possible that different aggregation states of proteins caused by phase separation could affect not only various chemical signals, but also mechanical activity.

Uneven distribution of intracellular mechanical activity is a prerequisite for cancer cell motility, which relies on some scaffold protein polarization or activation at membrane ruffling areas [75, 76]. Previous studies show that Rho GTPases, such as CDC42 and Rac1, are polarized and activated at membrane ruffling areas, accompanied by IQGAP1 translocation to the leading edge (Fig. 7A, B). Meanwhile, the RacGAP-C domain of IQGAP1 combines with the CAP-GLY domain of Clip170 and guides Clip170 tension to the cell cortex [76], resulting in a higher and faster Clip170 tension at the leading edge (Supplementary Fig. 1). Unlike IQGAP1, IQGAP2 is reported to inhibit intrinsic CDC42 activity [77], exerting a dominant-negative role in Clip170 force generation. Recent studies show that IQGAP2 binds directly to Clip170 and inhibited the IQGAP1–Clip170 interaction [78]. Similarly, our data indicate that *IQGAP2* overexpression eliminates the difference in Clip170 force between the leading edge and the following edge and *IQGAP2* knockdown enhances this difference (Fig. 8C). In summary, we suggest IQGAP1 as a vital regulator on Clip170 tension polarization and cancer invasion and migration.

Clip170 is considered to be a seed for F-actin assembly at MT plus ends [73], playing a vital role in the formation of cortactin-associated filopodia and lamellipodia (Fig. 7F, G). Therefore, we attempt to analyze the association between Clip170 tension with MT and MF force during directional migration. Firstly, Clip170 tension is polarized at the leading edge and guides MTs elongation towards the direction of cell movements. MTs stiffen the cytoskeleton, allowing it to resist higher compressive loads and regulate the direction of the invasive phenotype [10]. Thereafter, the forward forces, together with osmotic pressure, push the cytomembrane to form a membrane protrusion and the rigid MTs maintain the membrane protrusions [79]. Subsequently, cortical MF networks generate inward contraction and contribute to the alteration of the cell shape, which promotes filopodia and lamellipodia formation along the direction of MT elongation.

This study demonstrates that Clip170 tension is necessary for breast cancer aggressiveness, which relies mostly on the MT force and the motor molecule dynein. Clip170 phosphorylation promotes the conversion of a discrete comet-like Clip-170 distribution into a spotty pattern in response to RSK activation, resulting in increased Clip170 tension and the formation of filopodia and lamellipodia. The IQGAP1 subcellular distribution is also correlated with directional migration by controlling Clip170 tension polarization. Our study provides mechanical insights into breast cancer metastasis, and introduces Clip170 tension as a potential opportunity to advance breast cancer therapy.

## Supplementary information

Supplementary Figure legends

reproducibility checklist

sFigure 1.

sFigure 2.

sFigure 3.

sFigure 4.

sFigure 5.

Original Data File

Original Data File

## Data Availability

The datasets used and/or analyzed of this study are available from the corresponding author upon reasonable request.
