# Supplementary material for: Tension of plus-end tracking protein Clip170 confers directionality and aggressiveness during breast cancer migration
Source: Cell Death Dis. 2022 Oct 8;13(10):856. doi: 10.1038/s41419-022-05306-6 (PMC9547975; doi:10.1038/s41419-022-05306-6)
Supplement: Supplementary file 1 — Supplementary Figure legends [file 41419_2022_5306_MOESM1_ESM.docx]

**sFigure 1. Clip170 tension promote breast cancer aggressiveness**

(A) Fluorescence recovery after photobleaching (FRAP) was used to test the reliability of the Clip170-cpstFRET probe in cells. (B) FRET acceptor photobleaching analysis (FRET AB) was used to test the intracellular mobility of the Clip170-cpstFRET probe in cells. (C) Left panel: 15-min time-lapse images of MCF7 cells after Nocodazole treatment, Calibration bar, 0.2–2.0; scale bar, 10 μm. Right panel: Normalized CFP/FRET signals for the whole cell corresponding to Clip170 tension versus time (Mean±SEM，n≥6 cells in each group). (D) Left panel: 15-min time-lapse images of MCF7 cells after Ciliobrevin D treatment. Calibration bar, 0.2–2.0; scale bar, 10 μm. Right panel: Normalized CFP/FRET signals for the whole cell corresponding to Clip170 tension versus time (Mean±SEM，n≥6 cells in each group). (E) 15-min time-lapse images of MDA-MB-231 cells after CXCL12 treatment. Calibration bar, 0.2–2.0; scale bar, 10 μm. (F) Left panel: Pixel count distribution analysis of of CFP/FRET ratios in different ROI of MCF7 cells. Right panel: Normalized CFP/FRET signals for the whole cell corresponding to tension versus time. (G, I and K) Representative CFP and CFP/FRET images of directional migratory MDA-MB-231 (G), SKBR3 (I) and MDA-MB-468 (K) cells are presented. (H, J and M) Left panel: The pixel count distribution of CFP/FRET ratios in leading edge and follow edge. Right panel: The pixel count distribution of CFP/FRET ratios in leading edge and cell periphery. Calibration bar, 0.2–2.0; scale bar, 10 μm.

**sFigure 2. TTL regulates breast cancer progression via Clip170 tension**

(A) Western blotting analysis of detyrosinated-tubulin levels after *TTL* knockdown, *TTL* overexpression and *TTL* rescue in MDA-MB-468 cells. (B) 15-min time-lapse images of FRET analysis in MCF7 cells expressing the cpst-FRET tubulin probe and transfected with *TTL* siRNA and a *TTL* overexpression plasmid after CXCL12 treatment, Calibration bar, 0.2–2.0; scale bar, 10 μm. (C) CFP/FRET signals corresponding to tension versus distance. (D) Normalized CFP/FRET signals for the whole cell corresponding to tension versus time (Mean±SEM，n≥6 cells in each group). (E) Left panel: 15-min time-lapse images of FRET analysis in SKBR3 cells expressing the Clip170-cpstFRET probe and transfected with the *TTL* siRNA or *TTL* overexpression plasmid after CXCL12 treatment. Calibration bar, 0.2–2.0; scale bar, 10 μm. Right panel: Normalized CFP/FRET signals for the whole cell corresponding to tension versus time, respectively. (Mean±SEM, n≥6 cells in each group). (F) Left panel: 15-min time-lapse images of FRET analysis in MDA-MB-231 cells expressing the Clip170-cpstFRET probe and transfected with the *TTL* siRNA or *TTL* overexpression plasmid after CXCL12 treatment. Calibration bar, 0.2–2.0; scale bar, 10 μm. Right panel: Normalized CFP/FRET signals for the whole cell corresponding to tension versus time, respectively. (Mean±SEM, n≥6 cells in each group). (G) Flow cytometry detection of apoptosis in the control, *TTL* siRNA, *TTL* overexpression, and *TTL* rescue groups. (H) Crystal violet staining in the control, *TTL* siRNA, *TTL* overexpression, and *TTL* rescue groups. The proliferation activity is reflected by the cell numbers. (I) Representative images of the 3D spheroid invasion assays in the control, *TTL* siRNA, *TTL* overexpression and TTL rescue groups.

**sFigure 3. Clip170 phosphorylation promotes breast cancer progression both *in vivo* and *in vitro***.

(A) Western blotting analysis of CFP-Clip170 level in the WT and Clip170 mutation groups. (B) Flow cytometry detection of apoptosis in WT, S311A, S311D, S-A, and S-D groups. (C) Crystal violet staining in the WT, S311A, S311D, S-A, and S-D groups. The proliferation activity is reflected by the cell numbers. (D) Representative images of the 3D spheroid invasion assays in the WT, S311A, S311D, S-A, and S-D groups. (E) For the tail vein injection assay, bioluminescent images of systemic metastases in nude mice with Clip170-WT, Clip170-S311A, and Clip170-S311D MDA-MB-468 cells are shown. Bioluminescence calibration bar, 0.1 × 10^8^–2.5 × 10^9^ . (F) Representative images of the micro CT analysis of lungs from nude mice in tail vein injection group.

**sFigure 4. Clip170 phosphorylation regulates EMT in breast cancer**

(A) Representative images of long-time imaging analysis in WT and Clip170 mutation cells after TGFβ treatment, scale bar, 20 μm. (B) Quantitative analysis of the length-to-width ratio in each group (Mean±SEM, n≥10 cells in each group, ***P<0.001 compared with WT group). (C) Western blotting analysis of N-cadherin, E-cadherin, and vimentin levels in the WT and Clip170 mutation groups after TGFβ treatment.

**sFigure 5. IQGAP1 and 2 expression levels regulate Clip170 tension and breast cancer aggressiveness**

A) Left panel: 15-min time-lapse images of FRET analysis in MCF7 cells expressing the Clip170-cpstFRET probe and transfected with *IQGAP1*-siRNA and *IQGAP2*-siRNA after CXCL12 treatment. Calibration bar, 0.2–2.0; scale bar, 10 μm. Right panel: Normalized CFP/FRET signals for the whole cell corresponding to Clip170 tension versus time (Mean±SEM, n≥6 cells in each group). (B) Left panel: 15-min time-lapse images of FRET analysis in MDA-MB-468 cells expressing the Clip170-cpstFRET probe and transfected with *IQGAP1*-siRNA, *IQGAP2*-siRNA, the IQGAP2 overexpression plasmid, and *IQGAP2* rescue plasmid after CXCL12 treatment. Calibration bar, 0.2–2.0; scale bar, 10 μm. Right panel: Normalized CFP/FRET signals for the whole cell corresponding to Clip170 tension versus time (Mean±SEM, n≥6 cells in each group). (C) Representative images of the migration and invasion of the cells in the *IQGAP-*siRNA, *IQGAP2*-siRNA, *IQGAP2* overexpression, and *IQGAP2* rescue groups. (D) Quantitative analysis of the migration and invasion rates (Mean±SEM, n=3 independent experiments and at least three field, ***P<0.001 compared with NC group).
