# Supplementary figures and images for: Tension of plus-end tracking protein Clip170 confers directionality and aggressiveness during breast cancer migration
Source: Cell Death Dis. 2022 Oct 8;13(10):856. doi: 10.1038/s41419-022-05306-6 (PMC9547975; doi:10.1038/s41419-022-05306-6)

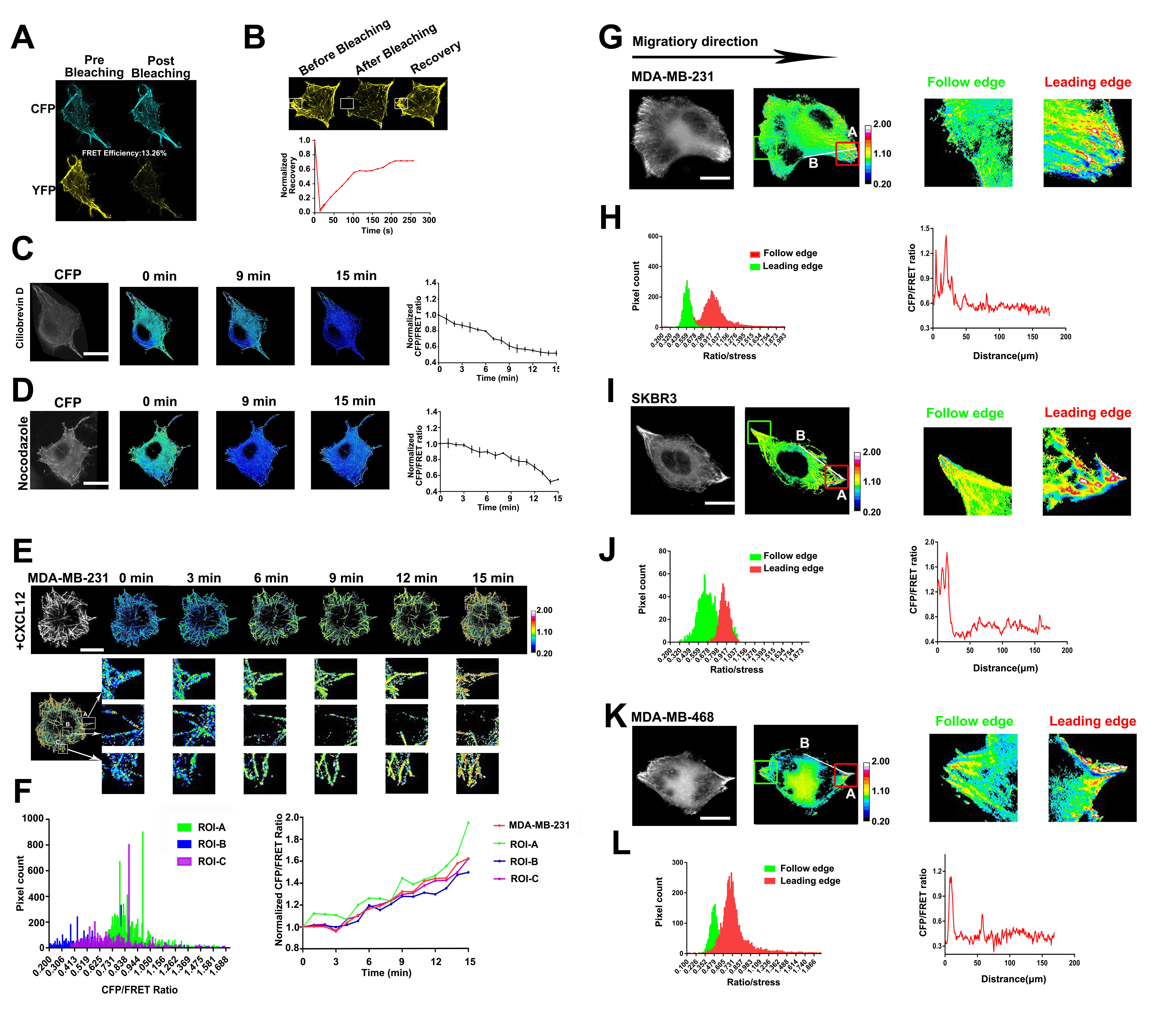

Supplement: Supplementary file 3 — sFigure 1. [file 41419_2022_5306_MOESM3_ESM.jpg]

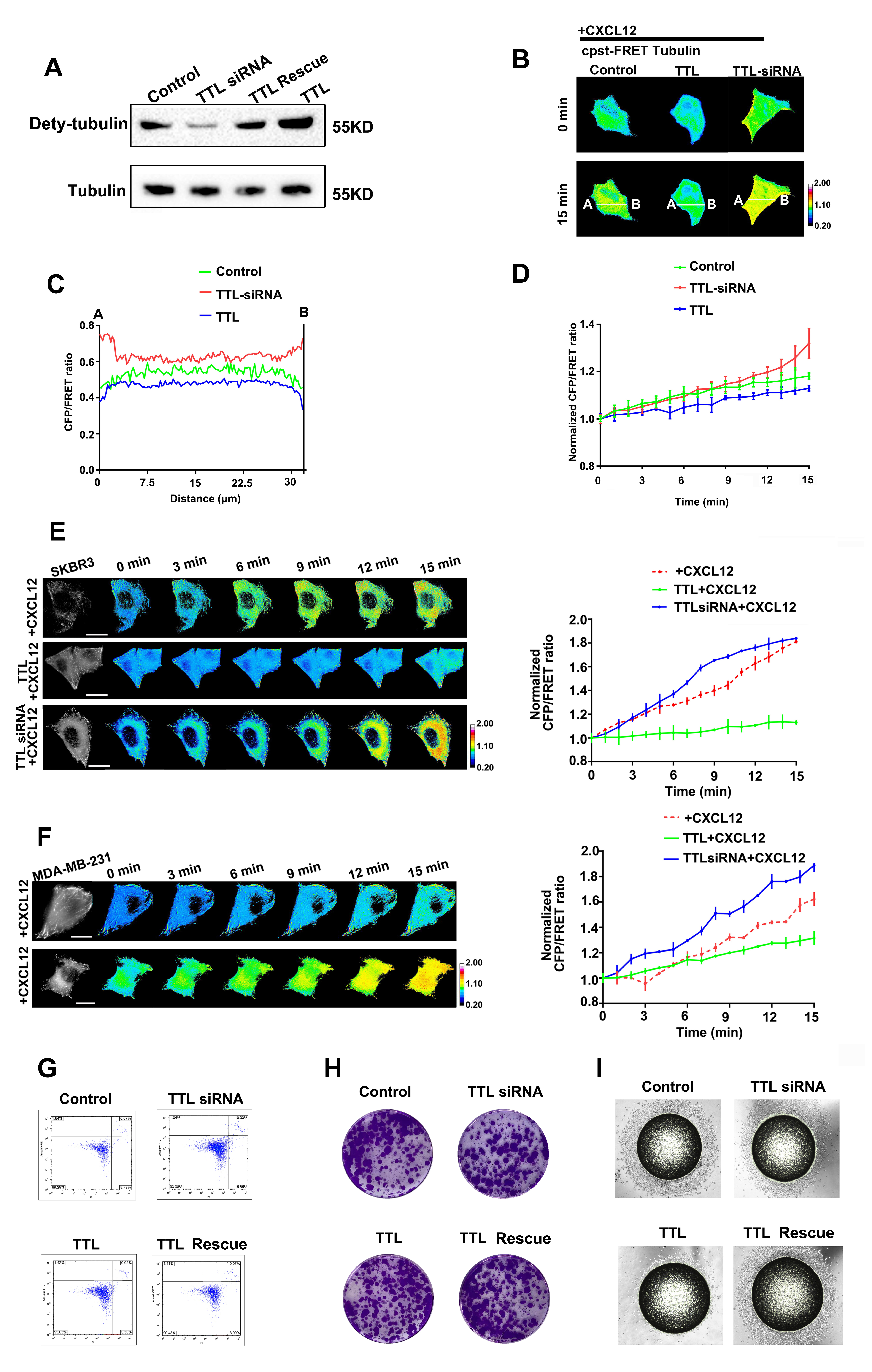

Supplement: Supplementary file 4 — sFigure 2. [file 41419_2022_5306_MOESM4_ESM.jpg]

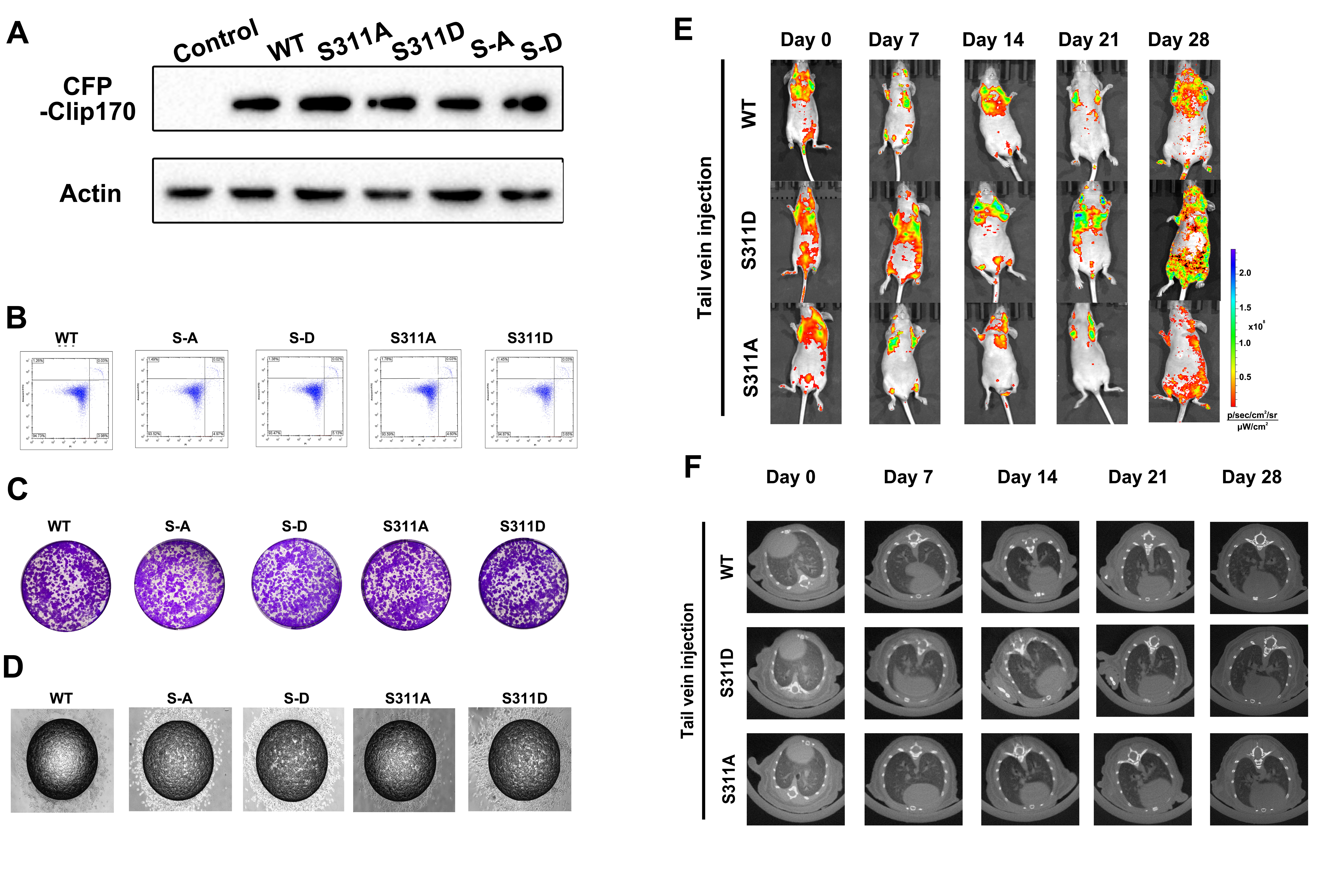

Supplement: Supplementary file 5 — sFigure 3. [file 41419_2022_5306_MOESM5_ESM.jpg]

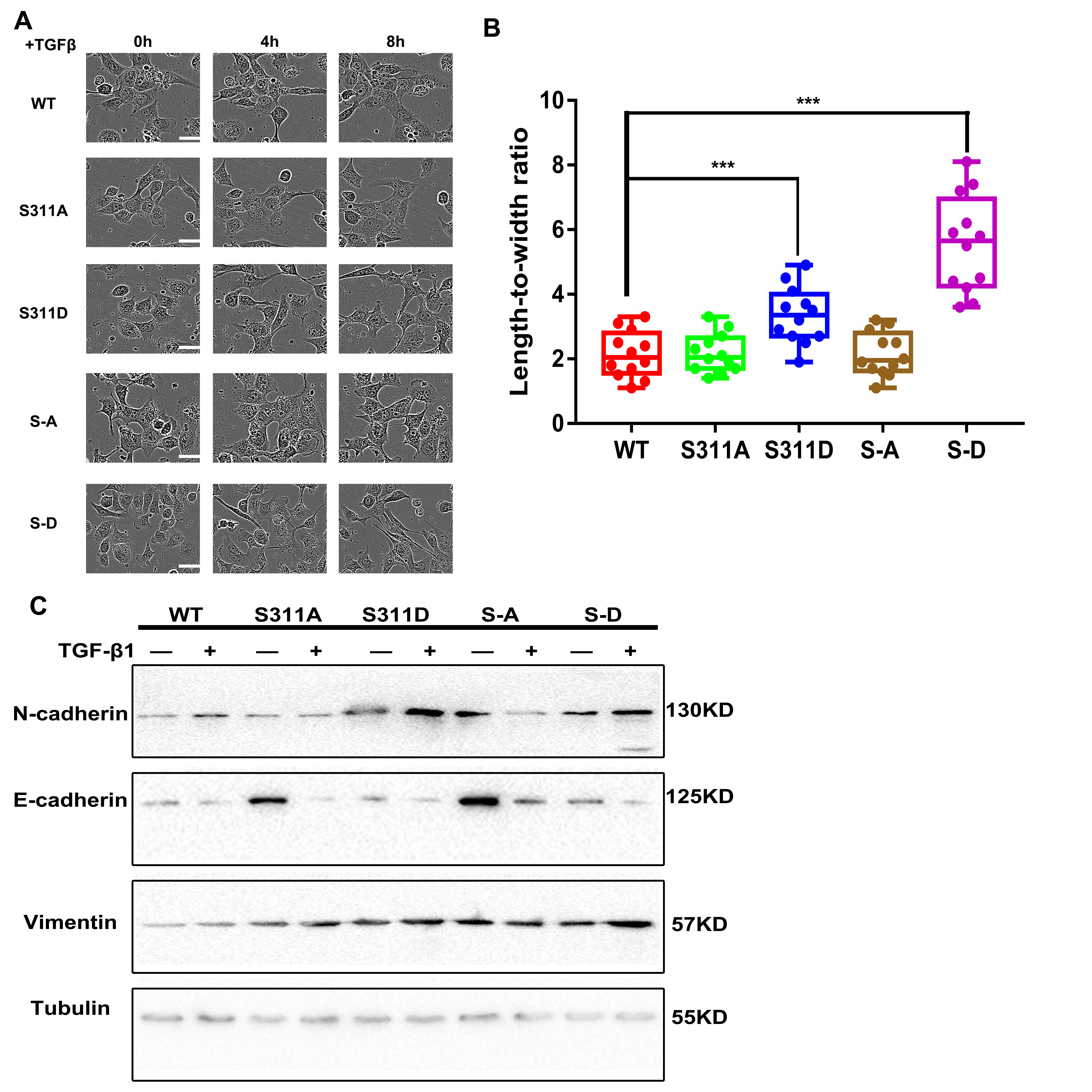

Supplement: Supplementary file 6 — sFigure 4. [file 41419_2022_5306_MOESM6_ESM.jpg]

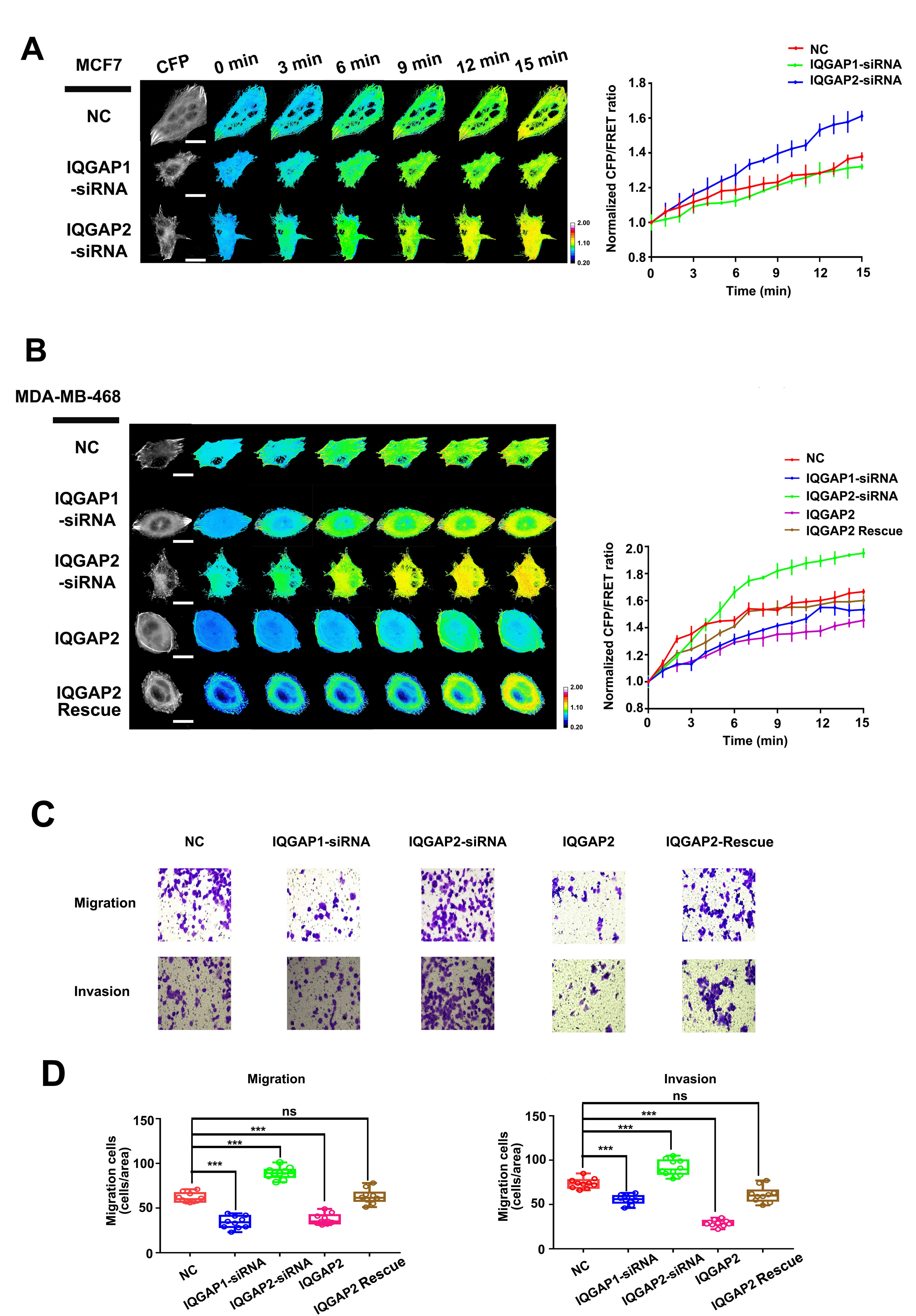

Supplement: Supplementary file 7 — sFigure 5. [file 41419_2022_5306_MOESM7_ESM.jpg]
