# Supplementary material for: Tension of plus-end tracking protein Clip170 confers directionality and aggressiveness during breast cancer migration
Source: Cell Death Dis. 2022 Oct 8;13(10):856. doi: 10.1038/s41419-022-05306-6 (PMC9547975; doi:10.1038/s41419-022-05306-6)
Supplement: Supplementary file 8 — Original Data File [file 41419_2022_5306_MOESM8_ESM.pdf]

Migration：

WT

Experiment 1: 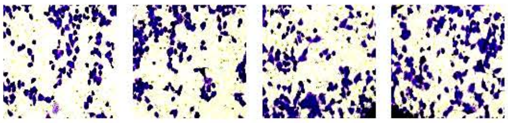

Experiment 2: 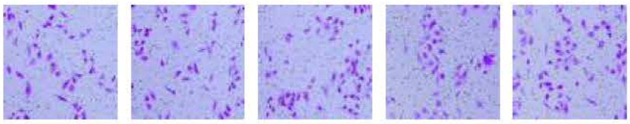

Experiment 3: 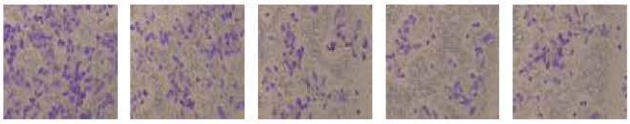

S-A

Experiment 1: 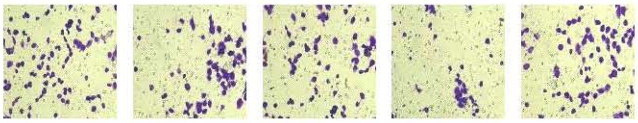

Experiment 2: 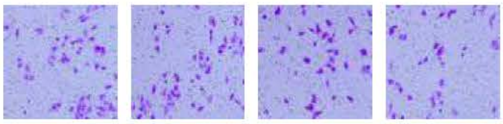

Experiment 3: 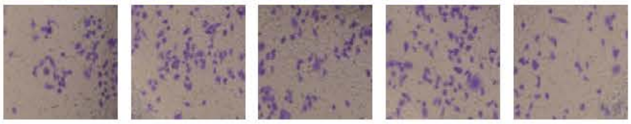

S-D

Experiment 1: 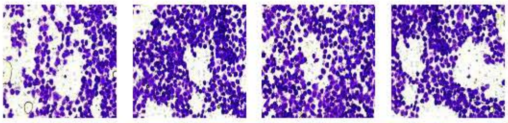

Experiment 2: 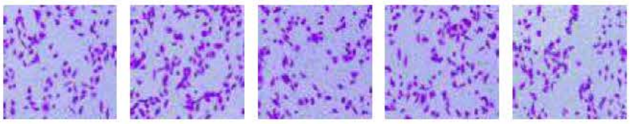

Experiment 3: 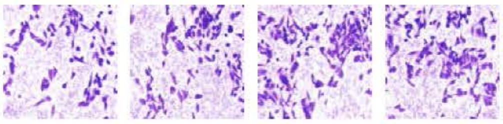

S311A

Experiment 1: 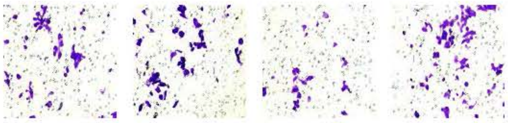

Experiment 2: 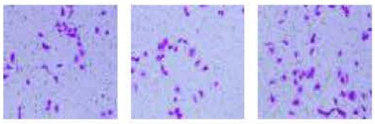

Experiment 3: 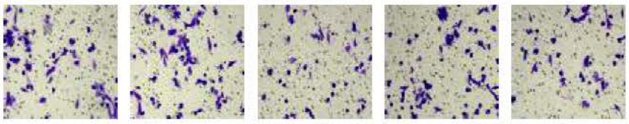

S311D

Experiment 1: 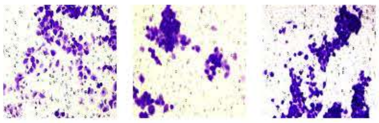

Experiment 2: 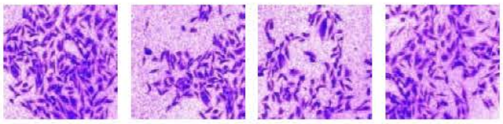

Experiment 3: 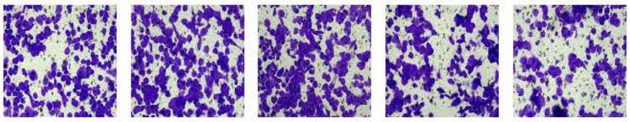

Invasion

WT

Experiment 1: 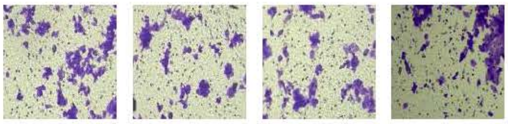

Experiment 2: 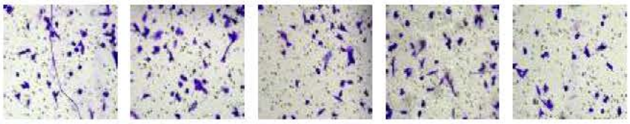

Experiment 3: 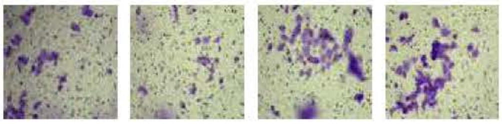

S-A

Experiment 1: 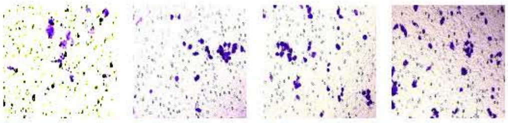

Experiment 2: 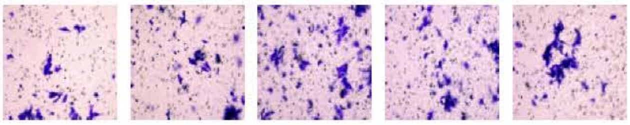

Experiment 3: 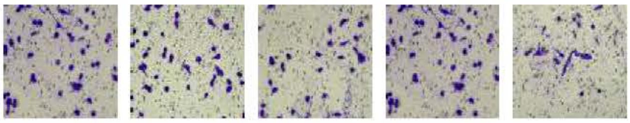

S-D

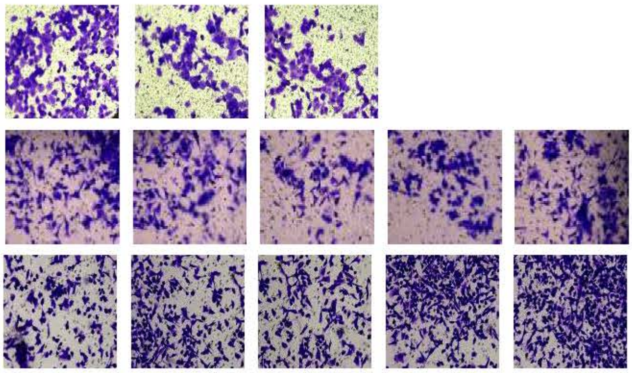

Experiment 1:

Experiment 2:

Experiment 3:

S311A

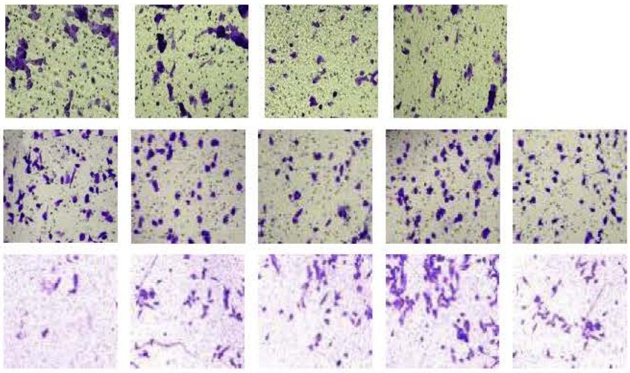

Experiment 1:

Experiment 2:

Experiment 3:

S311D

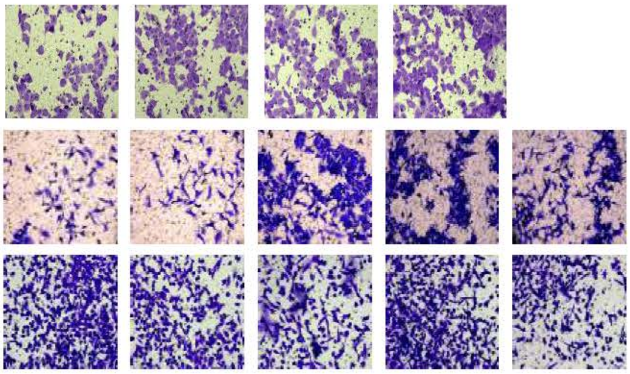

Experiment 1:

Experiment 2:

Experiment 3:
