# Supplementary material for: Tension of plus-end tracking protein Clip170 confers directionality and aggressiveness during breast cancer migration
Source: Cell Death Dis. 2022 Oct 8;13(10):856. doi: 10.1038/s41419-022-05306-6 (PMC9547975; doi:10.1038/s41419-022-05306-6)

Figure 3C

Clip170

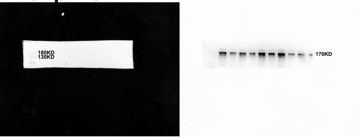

Clip170

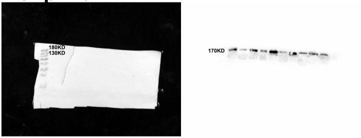

Detry-tubulin

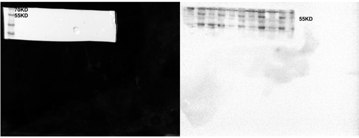

Detry-tubulin

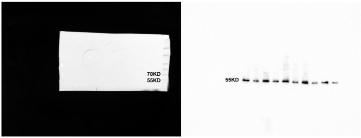

Tubulin

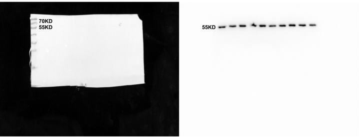

Tubulin

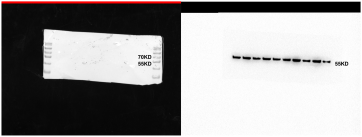

Figure 3D

Clip170+Detry-tubulin

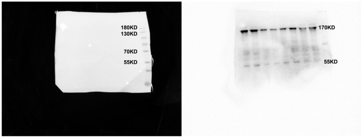

Tubulin

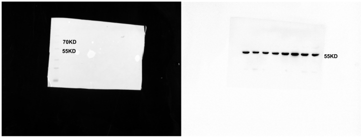

Supplementary Figure 2A

Detry-tubulin

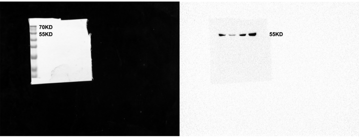

Tubulin

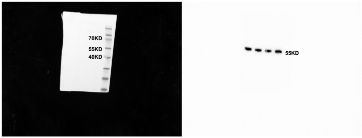

Figure 6D

RSK

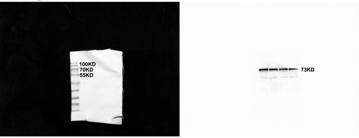

p-RSK

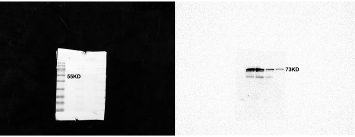

Tubulin

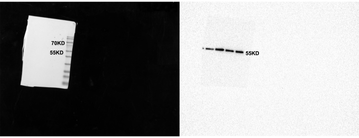

Figure 6E

RSK

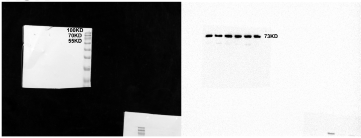

p-RSK

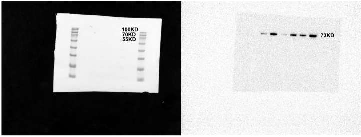

Tubulin

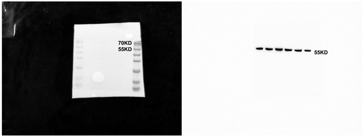

Figure 6I

RSK

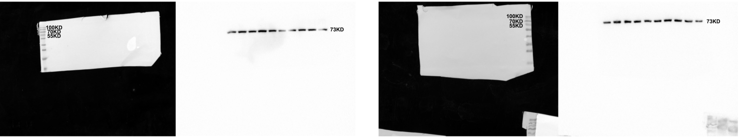

p-RSK

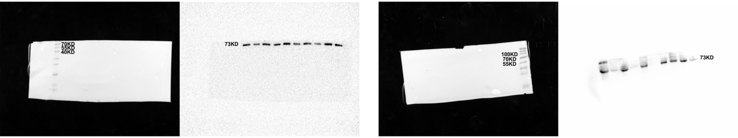

Tubulin

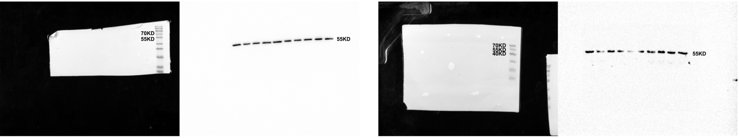

Figure 7C

IQGAP1

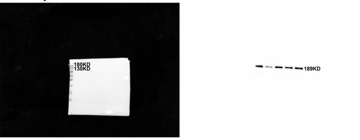

IQGAP2

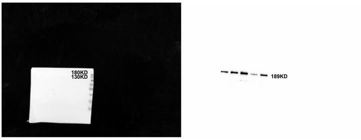

Cortactin

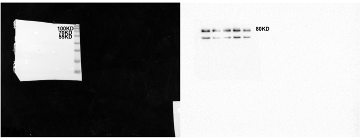

Tubulin

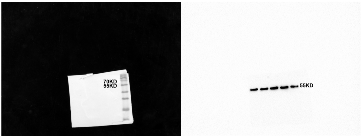

Figure 7D

Cortactin

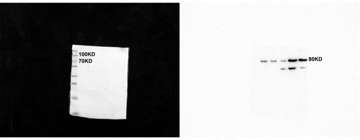

Tubulin

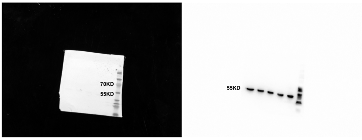

Figure 7E

Cortactin

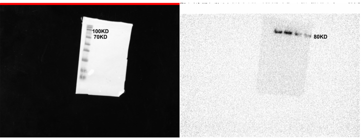

Tubulin

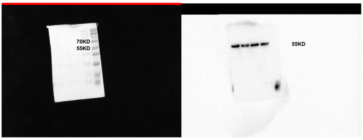

Supplementary Fig. 3A

CFP-Clip170

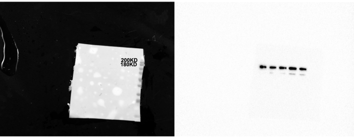

Actin

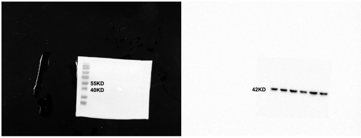

Supplementary Fig. 4C

N-cadherin

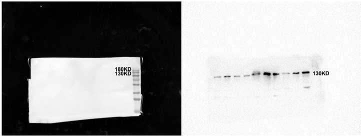

E-cadherin

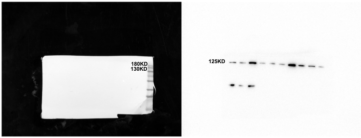

Vimentin

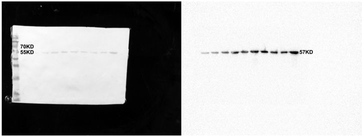

Tubulin

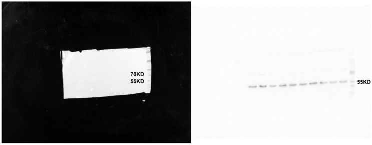

Supplement: Supplementary file 9 — Original Data File [file 41419_2022_5306_MOESM9_ESM.pdf]
